# Prognostic relevance of UCH-L1 and α-internexin in pancreatic neuroendocrine tumors

**DOI:** 10.1038/s41598-017-02051-1

**Published:** 2017-05-19

**Authors:** Yu-Li Song, Run Yu, Xin-Wei Qiao, Chun-Mei Bai, Chong-Mei Lu, Yu Xiao, Ding-Rong Zhong, Jie Chen, Yu-Pei Zhao, Tai-Ping Zhang, Tian-Tian Song, He-Li Gao, Ying-Hua Wan, Lin Shen, Jie Chen, Bin Lv, Jian-Jiang Hao, Ye Zhang, Laura Tang, Yuan-Jia Chen

**Affiliations:** 1 Department of Gastroenterology, Peking Union Medical College Hospital, Peking Union Medical College, Chinese Academy of Medical Sciences, Beijing, 100730 China; 2 Division of Endocrinology, Cedars-Sinai Medical Center, and Division of Endocrinology, Diabetes & Metabolism, UCLA, Los Angeles, California, 90095 USA; 3 Department of Oncology, Peking Union Medical College Hospital, Peking Union Medical College, Chinese Academy of Medical Sciences, Beijing, 100730 China; 4 Department of Pathology, Peking Union Medical College Hospital, Peking Union Medical College, Chinese Academy of Medical Sciences, Beijing, 100730 China; 5 Department of Surgery, Peking Union Medical College Hospital, Peking Union Medical College, Chinese Academy of Medical Sciences, Beijing, 100730 China; 6 Department of Gastrointestinal Medical Oncology, Peking University School of Oncology, Beijing Cancer Hospital and Institute, Beijing, 100142 China; 7 Department of Gastroenterology, the First Affiliated Hospital of Sun Yat-Sen University, Guangzhou, 510080 China; 8 Department of Gastroenterology, the First Affiliated Hospital of Zhejiang Chinese Medical University, Hangzhou, 310006 China; 9 Poochon Scientific, Frederick, MD 21704 USA; 10 Department of Molecular and Biochemistry, Institute of Basic Medical Sciences, Peking Union Medical College, Chinese Academy of Medical Sciences, Beijing, 100730 China; 11 Department of Pathology, Memorial Sloan-Kettering Cancer Center, New York City, 10065 USA

## Abstract

Prognostic biomarkers for the pancreatic neuroendocrine tumors are needed. Proteomic study on insulinoma has been rarely reported. We identified the differential expression of proteins between insulinoma and their paired tissues by proteomic analysis, and evaluated the prognostic significance of specific proteins in pancreatic neuroendocrine tumors including insulinoma. The differential expression of select proteins was validated in more than 300 tumors using immunohistochemical staining and western blot. Methylation of UCH-L1 promoter in tumors was examined by methylation specific PCR and validated by sequencing. The concurrent expression of UCH-L1 and α-internexin was correlated with the prognosis in 2 independent collectives of patients with tumors. Sixty-two and 219 proteins were significantly down-regulated and up-regulated in insulinomas, respectively. Demethylation of UCH-L1 promoter was associated with UCH-L1 expression in tumors (*p* = 0.002). The concurrent expression of UCH-L1 and α-internexin in pancreatic neuroendocrine tumors was significantly associated with better overall survival and disease-free survival in the combination of both cohorts (log rank *p* = 3.90 × 10^−4^ and *p* = 3.75 × 10^−5^, respectively) and in each of cohorts. The prognostic value of both proteins was also validated in patients with stage II and III tumors (*p* = 0.017 and *p* = 0.006, respectively). The proteins UCH-L1 and α-internexin could be independent prognostic biomarkers of pancreatic neuroendocrine tumors.

## Introduction

Pancreatic neuroendocrine tumors (PNETs) are uncommon with an incidence of 1–10 /million/year^1–7^. The incidence and prevalence of PNETs have increased over the past 30 years, not only in western countries but also in Asia^1, 3, 8–15^. Insulinoma is a main type of PNETs and causes hyperinsulinemic hypoglycemia^3, 16, 17^. The molecular alterations underlying insulinoma tumorigenesis are not well addressed^5, 17^. Islet β-cell specific ablation of the *MEN1* gene causes insulinoma in mice^18^; while frequent loss of heterogeneity (LOH) of *MEN1* gene is found in human insulinomas^19^, *MEN1* gene mutations are rarely found^20, 21^. Insights into the molecular alterations in sporadic insulinoma are crucial not only for deciphering tumorigenesis in insulinoma and other PNETs but also for discovering biomarkers of prognosis. The clinicopathological criteria for PNETs prognosis are improved considerably by the ENETS and WHO staging and grading systems. PNETs tend to relapse after resection, even if the tumors originally had lower stage and lower grade. Thus, molecular biomarkers are required for predicting relapse and prognosis of PNETs.

Recently, a number of molecular profiling studies on PNETs have been reported^21–28^; these studies revealed somatic mutation of some genes and abnormal expression of miRNA and message RNA in PNETs. These molecular alterations might play roles in the tumorigenesis of PNETs, and could be correlated with the prognosis of PNETs. However, proteomic study on sporadic insulinoma has been rarely reported.

We previously demonstrated that α-internexin was extensively expressed in PNETs and could be a novel prognostic biomarker for overall survival^29^. However, α-internexin could not be used as a marker for disease-free survival^29^. As tumor recurrence is the predominant cause of death in PNET, if molecular biomarkers could be identified to predict the relapse or the aggressive behaviours of PNET in an individual patient before the recurrence happens, the patient would benefit from more stringent surveillance and more aggressive antitumor therapy.

Therefore, the aims of the present study were to investigate the differential expression of proteins between sporadic insulinoma and paired pancreas by proteomic analysis and to examine if some proteins could be molecular prognostic biomarkers for insulinomas and other PNETs.

## Results

### Clinicopathological Characteristics of All Patients and Tumors

All PNETs studied were well-differentiated. The clinicopathological features of each tumor/patient were listed in detail in Supplementary Table S1, and summarized in Table 1. Of 306 patients, 103 (33.6%) underwent enucleations, 65 (21.2%) had either head, body or tail resection, 59 (19.3%) had tail resection and splenectomy and 56 (18.3%) underwent Whipple procedure; the surgical procedures were not well documented in 23 patients (7.5%). Two hundred and forty-seven patients were followed up (80.7%) and median time of follow-up was 68 months.

**Table 1:** Summary of Clinicopathological Features of PNET Patients.

Clinical Features of PNET Patients		Number = 306
Gender (%)	Male	130 (42.5)
	Female	176 (57.5)
Median age at surgery (range)	Male	51 (15–85)
	Female	47 (17–84)
PNET function-type (%)	Functional	177 (57.8)
	Non-functional	129 (42.2)
PNET subgroup (%)	Insulinoma	151 (49.3)
	Non-insulinoma	165 (50.7)
Metastasis (%)	No metastasis	233 (76.1)
	Metastasis	73 (23.9)
	LN metastasis only	42 (13.7)
	Distant metastasis	48 (15.7)
	Hepatic	41 (13.4)
	others	11 (3.6)
Grade (%) n = 252	G1	140 (55.6)
	G2	106 (42.1)
	G3	6 (2.4)
Stage (%) n = 305	I	95 (31.1)
	II a	98 (32.1)
	II b	34 (11.1)
	III	31 (10.2)
	IV	47 (15.4)
Follow-up information	Available	247 (80.7)
	Not available	59 (19.3)
Follow-up months, median (range)	68 (1–218)	
	Disease-free survival (DFS) (%)	170 (68.8)
	Alive with disease (AWD) (%)	27 (10.9)
	Died of disease (tumor) (DOD) (%)^a^	38 (15.4)
	Died of unknown cause (DUC) (%)	10 (4.0)
	Survival with unknown status (%)^b^	2 (0.8)
Clinicopathological features of tumors		Number = 314
Primary tumor location (%) n = 304	Pancreatic head	127 (41.8)
	Pancreatic body or tail	165 (54.3)
	Non-pancreas	12 (3.9)
Median tumor size (range) (cm) n = 302		2.5 (0.7–17)
Ki-67 (%) n = 235	≤2%	171 (72.8)
	>2%	64 (27.2)

^a^One patient died of disease (tumor) but the survival time was unknown.

^b^Two patients were alive but their disease status was unknown.

### Differential Expression of Proteins in Insulinomas and Bioinformatic Analysis

**Figure 1:**
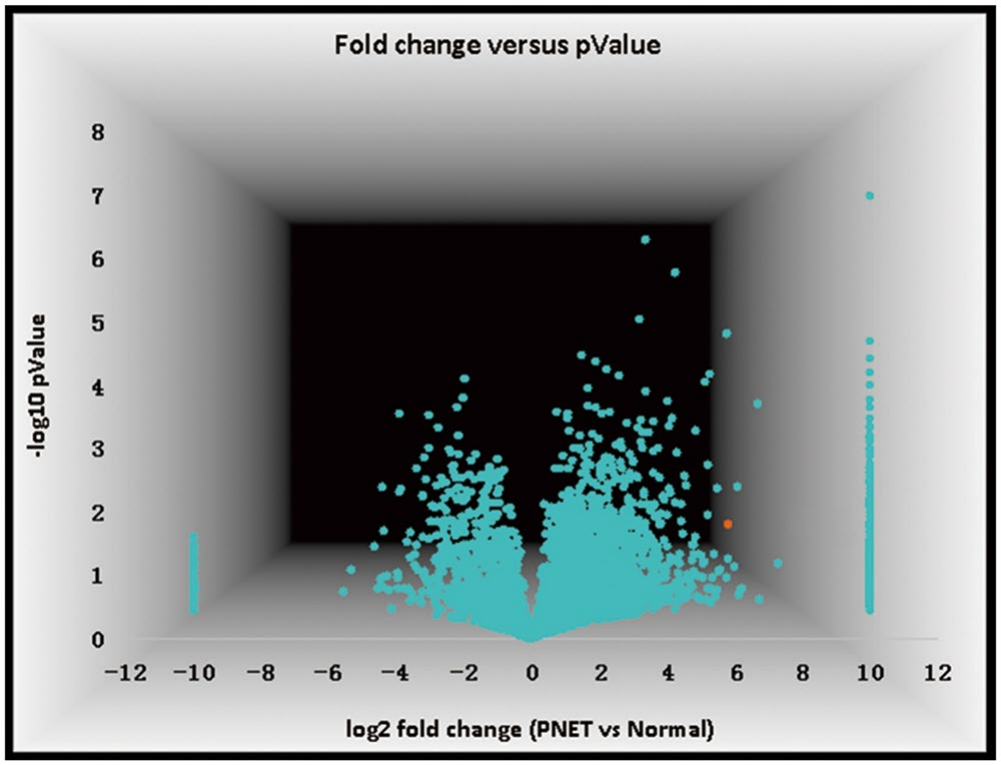
Volcano plot showing fold change of identified proteins between tumors and paired pancreatic specimens. The x-axis represents fold-changes of PNET versus para-tumor tissue (log2 of fold change), and the y-axis represents the statistical significance p-value (−log10 of p-value, n = 4). The orange dots represent UCH-L1.

Using quantitative proteomics approach, we assessed the global changes of the proteome by comparing the mean of relative abundance of proteins identified in 4 insulinomas with that of 4 paired pancreatic tissue samples. In this study, 5279 proteins were identified across all 8 samples, 3476 proteins were identified with more than two unique peptides (Supplementary Table S2). Quantitative analysis of the changes of the 3476 proteins between tumors and paired tissues revealed that 2021 proteins including housekeeping ones such as ribosomal proteins, GAPDH, tubulin were similarly expressed in both tumoral and paired tissues, while 1455 proteins were differentially expressed in tumor tissue and paired pancreatic tissue (Fig. 1). We identified that 219 of 1455 proteins were significantly up-regulated or expressed only in tumor tissues and 62 proteins were significantly down-regulated in tumor tissue or expressed only in paired pancreatic tissue. Among the 219 proteins which were up-regulated in tumor tissues, UCH-L1 was one of the most highly expressed proteins, the tumor/para-tumor ratio being 55.4, *P* = 0.016 (Fig. 1, the orange dot).

Bioinformatic analyses revealed that 24%, 15% and 11.6% of proteins were from cytoplasm, membrane and nucleus, respectively (Supplementary Fig. S1). These proteins were associated with a number of signal pathways, such as cytoskeleton signaling, protein ubiquitination pathway, VEGF signaling, mTOR and PI3K/AKT pathway (Supplementary Table S3).

### Validation of the Expressions of UCH-L1 and Other proteins in Subgroups of PNETs

**Figure 2:**
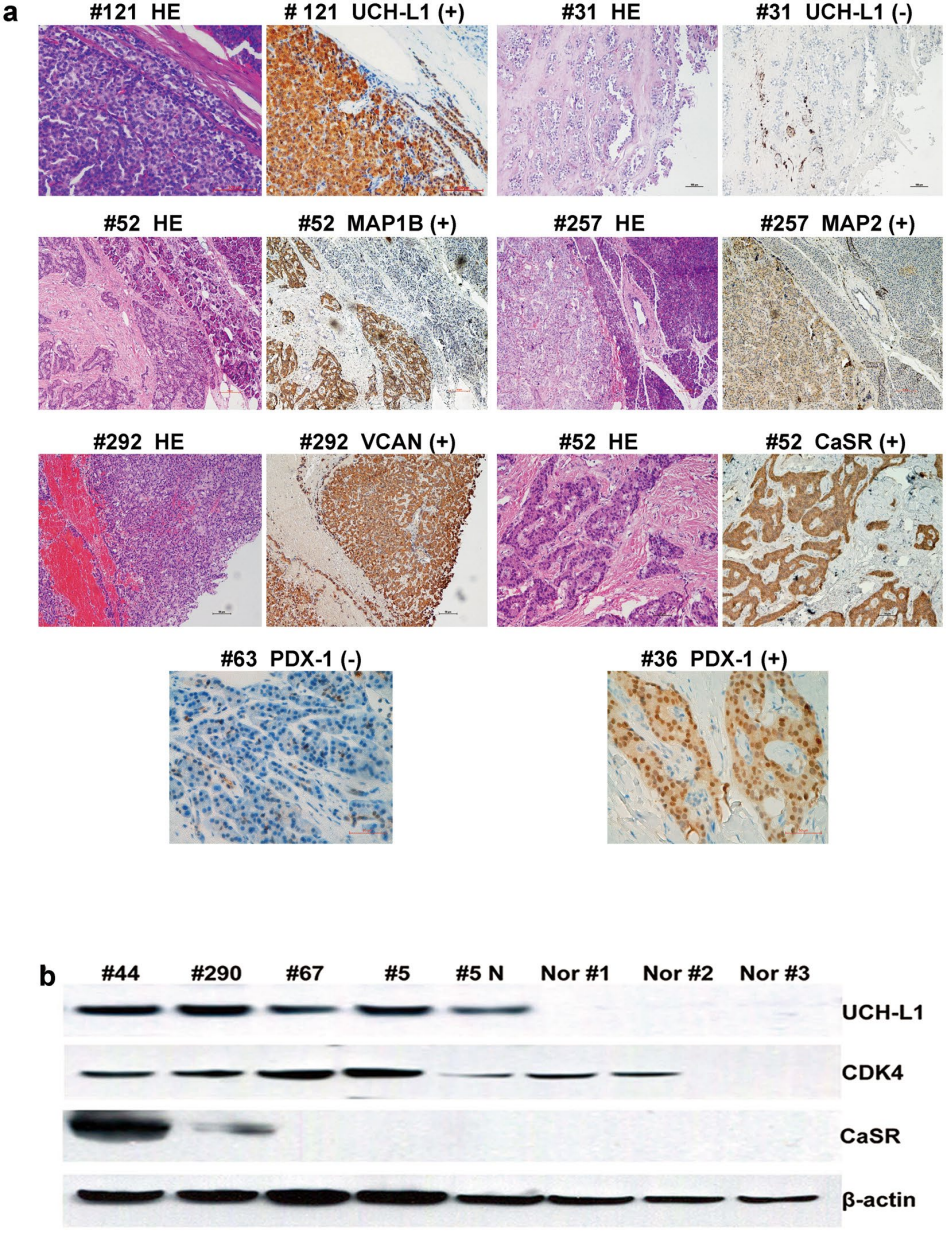
Representative examples of validated protein expression in PNETs and paired pancreatic specimens. Part **a**: IHC results. Left panel: HE staining of tumors and their paired pancreatic specimens; middle panel 1: expression of UCH-L1, MAP1B and VCAN in tumor #121, #52 (upper) and #292, respectively; middle panel 2: HE staining of tumors and their paired pancreatic specimens; right panel: negative expression of UCH-L1 in tumor #31 with a few positive cells, positive expression of MAP2 and CaSR in tumor #257 and #52, respectively; lower panel: expression of PDX-1 in insulinoma #36 and the negative expression of PDX-1 in tumor #63. Scale bar: 100 µm. Part **b**: Western blot results. Upper panel: expression of UCHL-1 in 4 PNETs, reduced expression of UCH-L1 in para-tumour tissue (#5 N) and no expression of UCH-L1 in 3 normal pancreatic specimens; upper middle panel 1: expression of CDK4 in 4 PNETs, 1 para-tumor tissue (#5 N) and 2 normal pancreatic specimens, no expression of CDK4 in 1 normal pancreatic specimen (Nor #3); lower middle panel 2: expression of CaSR in 2 PNETs and no expression of CaSR in 2 PNETs, para-tumor tissue (#5 N) and 3 normal pancreatic specimens; lower panel: β-actin was used as internal control. The identification of tumor was shown in Supplementary Table S1.

Differential expression of UCH-L1, MAP1B, MAP2, VCAN, CDK4 and PDX-1 was verified in more than 40 PNETs, including insulinomas and non-insulinomas by IHC but the expression of CaSR was only validated in 29 insulinomas (Supplementary Table S4 and Fig. 2). Furthermore, expression of protein UCH-L1, CDK4 and CaSR was confirmed by Western blot in 10 samples (Fig. 2b, Supplementary Table S4). By IHC, these proteins were extensively expressed in PNETs but were either not expressed or expressed at reduced levels in peritumoral tissues (Fig. 2a, Supplementary Table S4). PDX-1 was only expressed in 36 of 41 insulinomas. CDK4 was expressed in more than 98% of PNETs and 83% of peritumoral specimens (Fig. 2b, Supplementary Table S4). The biological and functional features of proteins UCH-L1, MAP1B, MAP2, VCAN, PDX-1, CDK4 and α-internexin were summarized in Table 2. Three of these proteins (MAP1B, MAP2 and α-internexin) are associated with cytoskeleton organization and PDX-1 is crucial for insulin expression^30^. In western blot analysis, intensity of protein band in each sample was quantified and listed in Table 3. For example, the signal of UCH-L1 in tumor # 5 (UCH-L1/β-actin = 0.342) was 8.3-fold stronger than that in its para-tumor tissue #5 N (UCH-L1/β-actin = 0.041) (Fig. 2b). A statistic analysis on ratio of UCH-L1/β-actin was performed. The ratio of UCH-L1/β-actin in tumor specimens was significantly higher than that in paired tissue or normal pancreatic specimens (*p* = 0.0095, Mann-Whitney U test, Table 3). The results from western blot were comparable to those from IHC. As UCH-L1 was one of the most highly expressed proteins in insulinomas and a number of studies showed that UCH-L1 was associated with biological behaviors in many types of tumors, we focused on UCH-L1 in the present study.

**Table 2:** Differentially expressed proteins between insulinomas and paired pancreatic issues.

	Proteins	NCBI accession	Tu/Nor ratio	PI	M_r_ (kDa)	Sequence Coverage (%)	Function by Gene Ontology Annotation Database	Process involved (experimental evidence) Gene Ontology Annotation Database
136681	UCHL-1: Ubiquitin carboxyl-terminal hydrolase isozymeL1	GI: 21361091	55.4	5.48	24.8	72.65	Ubiquitinbinding; protein binding; cysteine-type endopeptidase activity; omega peptidase activity	Negative regulation of MAP kinase activity; cell Proliferation; ubiquitin-dependent protein catabolic processetc.
317373388	MAP1B:Microtubule -associated protein 1B	GI:153945728	128.8	4.81	270.5	18.48	Protein binding; structural molecule activity	Microtubule organization; nervous system development; cellular process etc.
215274255	MAP2: Microtubule-associated protein 2	GI:87578396	>500	4.91	199.4	15.93	Dystroglycan, protein, microtubule, tubulin binding; structural molecule activity	Microtubule cytoskeleton organization; microtubule bundle formation; central nervous system neuron development etc.
2506816	VCAN: Versican core protein	GI: 21361116	54.2	4.51	372.6	5.92	protein binding	Cell adhesion; multicellular organismal development; central nervous system development; glial cell migration etc.
1708540	PDX-1: Pancreas/duodenum homeobox protein 1	GI:4557673	>500	7.56	30.8	12.01	Pranscription factor activity, sequence-specific DNA binding	Negative regulation of transcription from RNA polymerase II promoter; liver development; differentiation of pancreatic β cell; transcription, DNA-templated
1168867	CDK4: Cyclin-dependent kinase 4	GI:49457488	>500	7.01	33.7	10.89	Nucleotide, ATP, cyclin, protein complex binding etc.	Protein phosphorylation; circadian rhythm; positive regulation of cell proliferation etc.
20141266	INX: Alpha-internexin or NF-66	GI:14249342	33.4	5.4	55.4	18.84	Structural molecule activity; intermediate filament cytoskeleton, structural constituent of cytoskeleton	Multicellular organismal development; nervous system development; substantial nigra development; cell differentiation; organization

**Table 3:** Quantification of UCH-L1 protein expression in tumors and their paired tissues on western blot.

proteins	UCHL-1	CDK4	CaSR	β-actin	*Ratio of UCH-L1/β-actin	IHC result
intensity						
sample #						
#4	1450	/	/	1224	1.18	+
# 289	492	/	/	459	1.07	+
#44	660	155	1645	835	0.79	+
#290	713	290	15	880	0.81	+
#67	171	656	0	1335	0.13	+
#5	434	754	0	1267	0.34	+
#5 N	32	12	0	777	0.04	−
Nor #1	0	206	0	631	0	−
Nor #2	0	116	0	458	0	−
Nor #3	0	0	0	365	0	−

*The ratio of UCH-L1/β-actin in 6 tumor specimens (#4, #289, #44, #290, #67 and #5) was significantly higher than that in paired tissue (#5 N) or other normal pancreatic tissues (Nor 1, 2, 3), median 0.8 (0.13–1.18) vs. 0 (0–0.04), *p* = 0.0095, Mann-Whitney U test. The results from western blot are comparable with that from immunohistochemical (IHC) staining.

### Methylation of *UCH-L1* promoter in tumors

**Figure 3:**
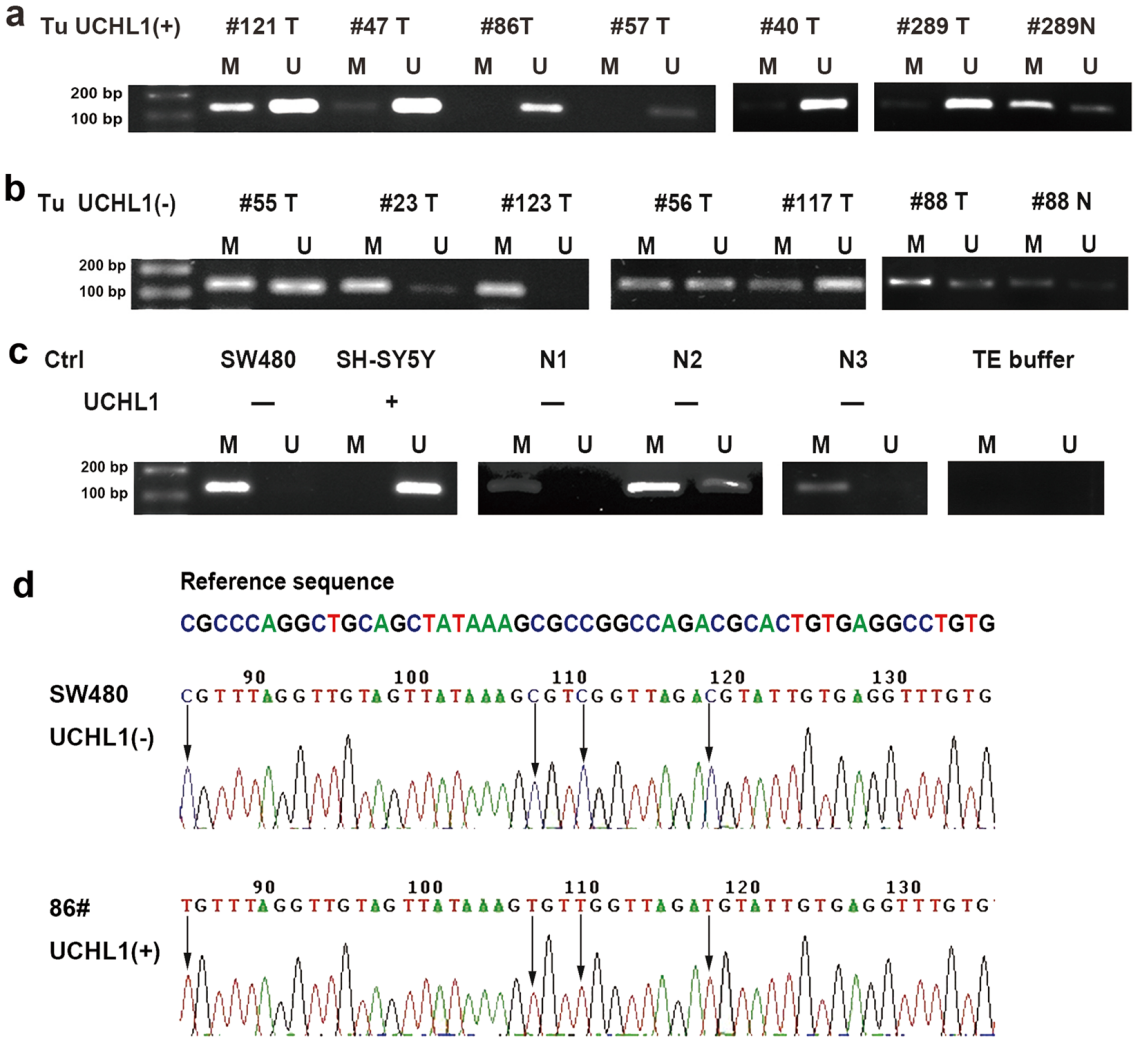
Promoter methylation of *UCH-L1* in cancer cell lines and PNETs. The methylation of *UCH-L1* promoter was more common in tumors without expression of UCH-L1 protein and para-tumor tissues (#289 N, #88 N). Demethylation was frequently seen in tumours with UCHL1 expression. U: unmethylated; M: methylated. Cell lines SW480 and SH-SY5Y were used as methylation and unmethylation controls, respectively, and TE buffer as blank control. Sequencing PCR products confirmed the MSP results.

It is reported that expression of the *UCH-L1* gene is mainly regulated by promoter methylation status in several non-endocrine tumors^31, 32^. To study the mechanisms underlying the differential expression of UCH-L1 in PNETs, we checked *UCH-L1* promoter methylation in PNETs. We examined the promoter methylation status of *UCH-L1* in 21 fresh frozen PNET specimens, 9 paired peritumoral tissue samples and 3 normal pancreatic tissues using MSP (Fig. 3a–c), and the results were confirmed by bisulfite sequencing (Fig. 3d). The methylation of *UCH-L1* promoter was found in 20 of 20 samples without UCH-L1 expression and in 3 of 13 samples with expression, respectively. Conversely, demethylation of *UCH-L1* promoter was found in 13 of 13 samples with UCH-L1 expression and in 9 of 20 samples without expression, respectively, *p* = 0.002 (Fisher exact test). As negative and positive controls of methylation status of the *UCH-L1*, cell lines SH-SY5Y and SW480 showed complete demethylation and methylation of *UCH-L1* gene promoter, respectively (Fig. 3c). TE buffer was used as blank control (Fig. 3c). The data suggested that hypo- or demethylation of the *UCH-L1* gene promoter was significantly associated with UCH-L1 protein expression in PNETs.

### Correlation of Clinicopathological Features/Prognosis with UCH-L1 Expression

When we evaluated the prognosis in PNET patients, 10 patients who died of unknown reasons were excluded. UCH-L1 expression was associated with disease-free survival in 104 patients with insulinomas (*p* = 0.047, Supplementary Table S5). Thus, we naturally extended our analysis to other PNETs, and we found that UCH-L1 expression was also associated with disease-free survival in 235 patients with PNETs (*p* = 0.013, Supplementary Table S5). UCH-L1 expression was also associated with less recurrence (*p* = 0.071, Supplementary Table S5). Moreover, Kaplan-Meier analysis showed that UCH-L1 expression in patients of collective I was associated with better overall survival (*p* = 0.034, Supplementary Fig. S2A) and disease-free survival (*p* = 0.019, Supplementary Fig. S2B). Multivariate analysis (Cox model), however, revealed that the UCH-L1 expression in PNETs was not associated with overall survival but still correlated with disease-free survival (Supplementary Table S6-a, model A, *p* = 0.463 and 6-b, model A, *p* = 0.018, respectively). These data suggested that UCH-L1 could be an independent prognostic marker of disease-free survival but not for overall survival.

### Correlation of Clinicopathological Features/Prognosis with UCH-L1 and α-internexin Expression

**Figure 4:**
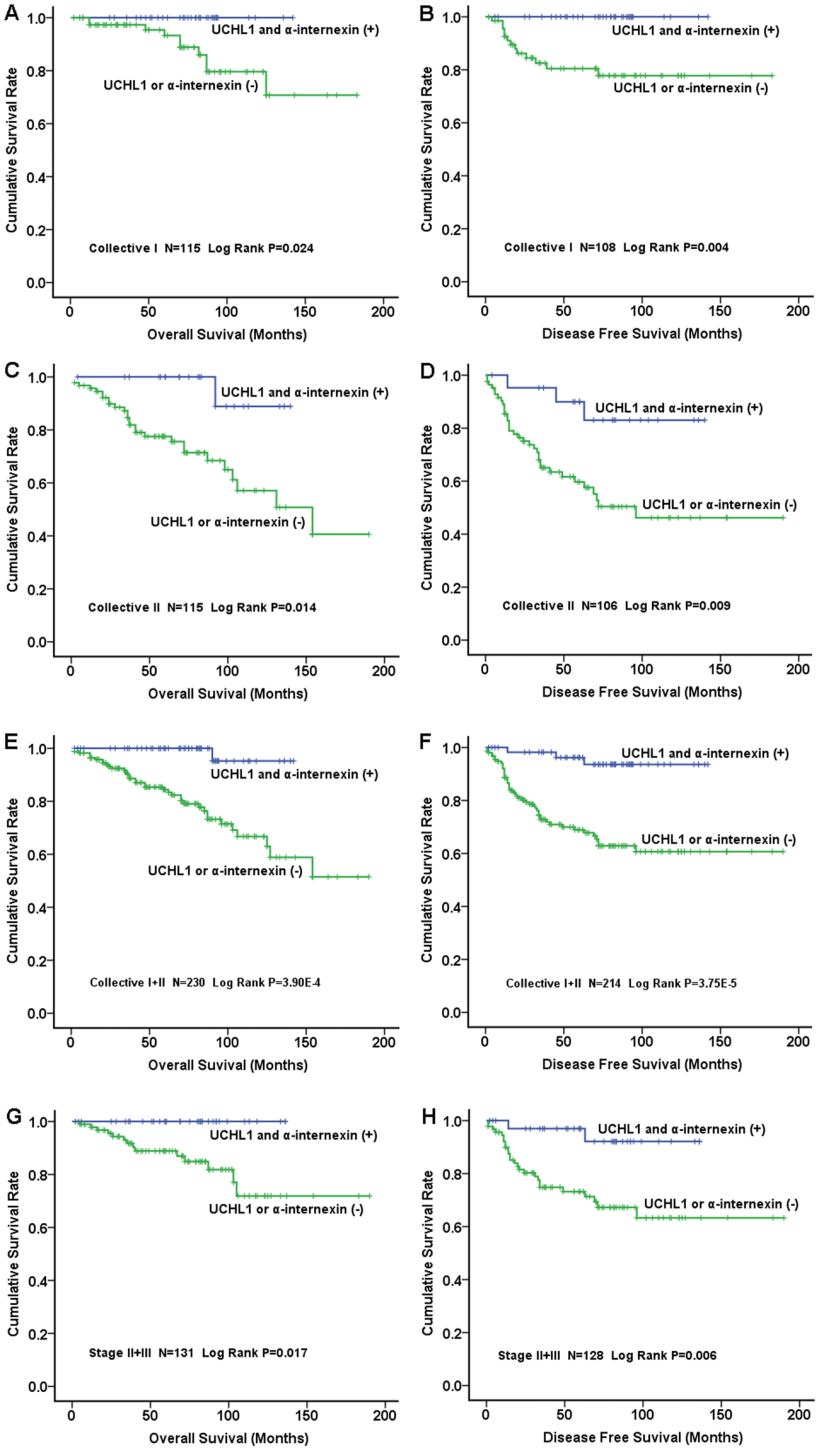
Correlation of Simultaneous Expression of UCH-L1 and α-internexin with Prognosis in 2 Collectives of Patients. Left panel: overall survival; right panel: disease free survival. The simultaneous expression of UCH-L1 and α-internexin in tumors was correlated with better overall survival and disease free survival in collective I (**A** and **B**, respectively) and a better overall survival with a statistical trend in collective II (**C**) and favorable disease free survival in collective II (**D**) as well as in the combination of collective I + collective II (**E** and **F**). The expression of both proteins in tumors was significantly associated with favourable overall survival (**G**) and disease free survival (**H**) in patients with stage II and III PNETs.

In our recent study on PNETs^29^, we found that the expression of α-internexin was significantly associated with overall survival but not with disease-free survival. We hypothesized that combination of both UCH-L1 and α-internexin might be useful in evaluating both overall survival and disease-free survival. The concurrent expression of UCH-L1 and α-internexin significantly correlated with better overall survival and disease-free survival in collective I (Fig. 4A, *p* = 0.024 and Fig. 4B, *p* = 0.004, respectively). Similarly, in collective II, the patients with concurrent expression of both proteins had a favorable overall survival (Fig. 4C, *p* = 0.014) and a better disease-free survival (Fig. 4D, *p* = 0.009). In the combination of both collectives, the concurrent expression of UCH-L1 and α-internexin proteins significantly correlated with a better overall survival (Fig. 4E, *p* = 3.90 × 10^–4^; Cox analysis: HR 0.141, 95% CI 0.018 to 1.088, *p* = 0.060, see Supplementary Table S6-a, model B) and a better disease-free survival (Fig. 4F, *p* = 3.75 × 10^−5^, Cox: HR 0.215, 95% CI 0.064 to 0.716, *p* = 0.012, see Supplementary Table S6-b, model B). The data suggested that concurrent expression of UCH-L1 and α-internexin could be an independent prognostic biomarker of PNETs.

Interestingly, we found that the concurrent expression of UCH-L1 and α-internexin in patients with stage II and III was significantly associated with better overall survival (Fig. 4G, *p* = 0.017) and disease-free survival (Fig. 4H, *p* = 0.006, Cox analysis: HR 0.167, 95% CI 0.038 to 0.731, *p* = 0.017, Supplementary Table S7).

The cross-sectional analysis was consistent with the findings revealed by Kaplan-Meier analysis and Cox’s proportional hazard model. In the combination of both collectives, the concurrent expression of both proteins was significantly associated with lower stages (*p* = 9.26 × 10^−5^), less recurrence (*p* = 4.53 × 10^−6^) and smaller tumor size (*p* = 1.79 × 10^−5^) (Table 4). Notably, only one of 60 patients (1.7%) with the expression of both proteins was dead while 37 of 170 patients (21.8%) whose tumors were without the expression of both proteins died of disease (*p* = 7.79 × 10^−5^, Table 4). In addition, 57 of 60 patients (95%) with the concurrent expression of both proteins had disease-free survival whereas 106 of 168 patients (63%) without concurrent expression of both proteins had disease-free survival at the last follow-up (*p* = 6.10 × 10^−7^, Table 4).

**Table 4:** Correlation of Clinicopathological Characteristics with Expression of UCH-L1 and α-internexin.

Clinicopathological Features	Expression of UCH-L1 and α-internexin		P value
	Present (%)	Absent (%)	
**Patients with PNETs (n** = **283)**	79 (27.9)	204 (72.1)	
age at diagnosis (year) (n = 283)	44.0 (19–79)	51.0 (16–85)	0.001
gender (n = 283)			
Male	32 (26.7)	88 (73.3)	0.688
Female	47 (28.8)	116 (71.2)	
grade (n = 243)			
G1	34 (24.8)	103 (75.2)	0.331
G2	27 (27.0)	73 (73.0)	
G3	0 (0)	6 (100)	
stage (n = 283)			
I	36 (41.4)	51 (58.6)	9.26 × 10^−5^
II a	31 (34.8)	58 (65.2)	
II b	5 (15.6)	27 (84.4)	
III	4 (13.3)	26 (86.7)	
IV	3 (6.7)	42 (93.3)	
recurrence (n = 229)			
No	57 (34.5)	108 (65.5)	4.53 × 10^−6^
Yes	4 (6.3)	59 (93.7)	
overall survival (n = 192)	59 (30.7)	133 (69.3)	7.79 × 10^−5^
death (n = 38)	1 (2.6)	37 (97.4)	
disease free survival (n = 163)	57 (35.0)	106 (65.0)	6.10 × 10^−7^
survival with disease or death (n = 65)	3 (4.6)	62 (95.4)	
**All PNETs (n** = **290)**			
location (n = 271)	79 (27.2)	211 (72.8)	
pancreatic head	37 (31.1)	82 (68.9)	0.323
body/tail	39 (25.7)	113 (74.3)	
Ki-67 (n = 223)			
≦2%	45 (27.4)	119 (72.6)	0.580
>2%	14 (23.7)	45 (76.3)	
Metastasis (n = 290)			
Yes	7 (8.9)	72 (91.1)	1.69 × 10^−5^
No	72 (34.1)	139 (65.9)	
size [range] (cm) (n = 281)	2 [1–15]	3 [0.7–17]	1.79 × 10^−5^

**Figure 5:**
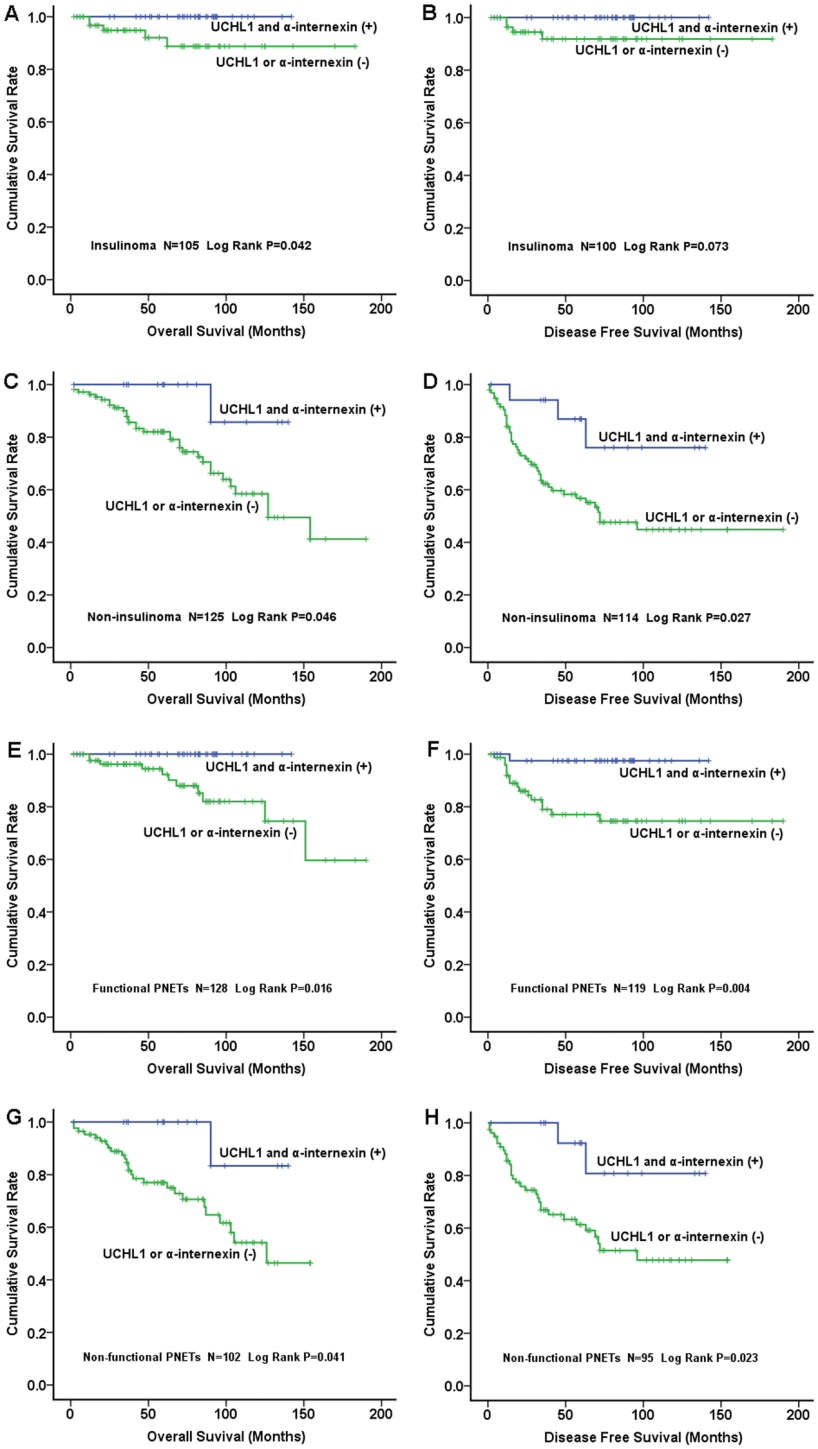
Correlation of Concurrent Expression of UCH-L1 and α-internexin with Prognosis in Subgroups of PNETs. Left panel: overall survival; right panel: disease-free survival. The simultaneous expression of UCH-L1 and α-internexin was correlated with better overall survival and disease-free survival in either insulinomas (Fig. 5A and B, respectively) or non-insulinomas (Fig. 5C and D, respectively). Similarly, the concurrent expression of both proteins was significantly associated with favourable overall survival and disease-free survival in either functional PNETs (Fig. 5E and F, respectively) or NF (Fig. 5G and H, respectively).

We also analyzed the prognostic value of the two proteins in subgroups of PNETs. Interestingly, the simultaneous expression of UCH-L1 and α-internexin in insulinomas was correlated with better overall survival of patients (Log rank, p = 0.042, Fig. 5A) but insignificantly with better disease-free survival (Log rank, *p* = 0.073, Fig. 5B). The simultaneous expression of UCH-L1 and α-internexin in patients with non-insulinomas was associateded with better overall survival and disease-free survival (Log rank, *p* = 0.046 and *p* = 0.027, Fig. 5C and D, respectively). Similarly, concurrent expression of both proteins was significantly associated with favourable overall survival and disease-free survival in patients with functional PNETs (Log rank, *p* = 0.016 and *p* = 0.004, Fig. 5E and F, respectively) or with NF (Log rank, *p* = 0.041 and *p* = 0.023, Fig. 5G and H, respectively).

Using multivariate analysis (Cox’s proportional hazard model), the concurrent expression of both protein is not significantly associated with better prognosis in each of subgroup of PNETs, although a statistical trend could be seen (p value between 0.150 to 0.08, see Supplementary Table S8), likely due to the smaller sample sizes when we divided all PNETs into 4 groups.

## Discussion

Tumor biomarkers have been used for diagnosis, management and prognosis by predicting tumor behavior or monitoring response to treatment. The biomarkers can be DNA, RNA and proteins; for example, high Akt expression could predict a nonresponse to chemotherapy in gastro-entero -pancreatic neuroendocrine tumors (GEP-NETs)^33^, over-expression of FGF13 mRNA is an independent predictor of a shorter progression-free survival^23^ and expression of KIT protein is an independent prognostic marker of mortality of PNETs^34^ and high expression of CD68 protein correlates with nonfunctional PNETs recurrence^35^.

A previous gene expression profiling study on human insulinoma found a number of genes that might play an important role in the pathogenesis of insulinoma^36^. Whole exome sequencing of insulinoma revealed a hotspot mutation of *YY1* gene that could play a role in the tumorigenesis of insulinoma^21^. Both studies, however, did not identify applicable molecular markers for evaluating prognosis of insulinomas. Recently, a study showed that CUX1 mediates progression and angiogenesis in murine neuroendocrine tumors and is associated with malignant behaviors in human insulinomas^37^. Another interesting study revealed that TPD52 protein was associated with survival of patients with insulinomas using proteomic approach^27^. It has not been addressed whether these proteins could be used as a prognostic marker in other subtypes of PNETs. The expression profiling study on PNETs found that down-regulation of PTEN or TSC2 correlated with tumor aggressiveness but as addressed by the authors, PTEN and TSC2 might not be independent prognostic biomarkers at multivariate analysis^23^. Thus, more reliable independent prognostic biomarkers for PNETs are needed to predict unfavorable outcome in a patient with PNET so that the patient would benefit by receiving individualized treatment and surveillance based on prognostic markers.

Mass spectrometry brings higher accuracy and detection capability, and allows simultaneous detecting of thousands of proteins in a single study, thereby leading to discovering novel and more-reliable biomarkers for tumors^27, 38^ or potential therapeutic targets^33^. Here, we found more than 200 proteins differentially expressed in insulinoma by mass-spectrometry-based proteomic analysis and validated the expressions of several proteins not only in insulinomas but also in PNETs as a whole. In the present study, we used para-tumor tissue as the control, which contains both islets and exocrine tissue, based on previous studies demonstrating that non-islet pancreas was an important origin of PNETs. For example, a NIH study using a combination of genetic and morphological analysis strongly suggested that PNETs are derived from the ductal/acinar system^39^. Perren *et al*. also revealed that MEN1-associated PNETs arise from at least two different cell compartments of the pancreas, i.e. islets and exocrine ducts^40^. It is not surprising that PNETs might originate from either endocrine or exocrine cellular compartment because β cells could differentiate from nonendocrine epithelial cells (duct and acinar cells) in the human pancreas^41^. Para-tumor tissue has been used as control for PNETs by others with meaningful results^22^. We thus believe that para-tumor tissue is an appropriate control in our proteomic analysis of PNETs.

The present proteomic study identified the differential expression of proteins between tumor tissue and paired pancreatic tissue while Alkatout *et al*. compared the protein expression between benign insulinoma and malignant insulinoma^27^. This is the major difference between the 2 proteomic studies on insulinomas. Alkatout *et al*. revealed a number of protein differentially expressed between benign and malignant tumors, for example, expression of GSN and TPD52 proteins was upregulated in benign insulinomas, both by 3.9 fold^27^. Interestingly, we also found expression of GSN and TPD52 was upregulated in benign insulinomas, by 4.3 and 3.7 fold, respectively.

Our previous study demonstrated that α-internexin, a component of cytoskeleton, is extensively expressed in PNETs and could be a novel prognostic biomarker for overall survival^29^. In our proteomic analysis, UCH-L1 was one of the proteins that were most strongly expressed in insulinomas. Our data showed that UCH-L1 might be an independent prognostic marker of disease-free survival in PNETs but not for overall survival. Thus, it was conceivable to combine UCH-L1 with α-internexin to assess their prognostic value in PNETs. As we demonstrated, the concurrent expression of both proteins proves valuable for predicting either disease-free survival or overall survival in each collective as well as in the combination of both cohorts. Moreover, the patients whose tumor expressed both proteins have lower probability of recurrence in each cohort and in the combination of both cohorts.

Another advantage of present study was that we assessed the prognostic value of these 2 proteins in a subgroup of patients with tumor stage II and stage III. Among 1072 patients with PNETs from 8 European cancer centers, only 1 of 248 patients with tumor stage I (ENETS staging) died of disease, in contrast, almost half of patients with tumor stage IV died of diseases^42^. In our most recent study on 977 PNETs patients^43^, we found that all of the patients with tumor stage I were alive without disease but more than 90% of patients with tumor stage IV died of disease or alive with tumor at the last follow-up. In fact, most patients with stage IV PNETs died of the disease eventually. Thus, it is more needed to predict the prognosis in patients with stage II and III than that in patients with stage I (very favorable outcome) or stage IV (the worst prognosis). However, few studies previously focused on PNET patients with tumor stage II and stage III. Our present data showed that concurrent expression of UCH-L1 and α-internexin could be prognostic biomarker for PNETs of stage II and stage III.

It is also interesting to note that the concurrent expression of both proteins might be of prognostic value in subtypes of PNETs, e.g. NF/functional PNETs or insulinoma/non-insulinoma although the correlation of concurrent expression of both proteins with survival does not reach the significance by using multivariate analysis. The multivariate analysis (Cox’s model) showed only a statistical trend between the concurrent expression of UCH-L1 and α-internexin and prognosis in each of the subgroups of PNETs (*p* value between 0.15 to 0.08), it is likely due to the sample sizes correspondingly reduced after we divided all PNETs into 4 groups.

UCH-L1 belongs to a deubiquitinases family which plays important roles in various cancer and may be potential therapeutic targets^44^. A number of studies showed that UCH-L1 was associated with many types of cancers including colorectal carcinoma^45^ and prostate cancer^46^. However, whether UCH-L1 inhibits tumor growth and tumor progression is controversial. The expression of UCH-L1 in tumor cells enhances their invasive potential *in vitro* and *in vivo* by regulating cell adhesion through Akt-mediated pathway, suggesting that the protein is an upstream regulator of Akt^47^. Some studies also showed that expression of UCH-L1 contributed to cell malignant transformation, tumor growth, metastasis and worse prognosis^46, 48, 49^, suggesting UCH-L1 is an oncogene product^50^. In contrast, other researchers find that expression of UCH-L1 induces apoptosis in breast cancer^51^ and, UCH-L1 knockdown in ovarian cancer cell lines caused increased proliferation^44^, suggesting that UCH-L1 is a tumor suppressor gene in these endocrine tumors. Two other studies also suggested UCH-L1 was a tumor suppressor in nasopharyngeal carcinoma and liver cancer^31, 52^. Hence, it seems that function of UCH-L1 gene (protein) and its clinical implication maybe tumor-type dependent.

Recently, an interesting study showed UCH-L1 expression in a group of well-differentiated GEP-NETs and the protein level was significantly higher among localized GEP-NETs compared with metastatic tumors^53^. The study suggested that loss of UCH-L1 expression was an independent risk factor associated with metastatic NETs at the surgery, which was consistent with our present finding that UCH-L1 expression was significantly associated with localized PNETs. That study, however, included only 11 PNETs and was unable to analyze the prognosis due to the small number of cases^53^. Moreover, UCH-L1 expression was only positive in 40% of cervical neuroendocrine carcinomas, a highly aggressive NETs^54^. In the present study, we found that combination of UCH-L1 with α-internexin could be better and more reliable for evaluating prognosis in PNETs than using single protein biomarker because expression of UCH-L1 alone was associated with disease-free survival only, whereas α-internexin was only correlated with overall survival.

Our study has some limits. At least half of patients were followed up retrospectively; the limitations of retrospective study could exist in the study, such as the variability in different hospitals, tissue availability might lead to the potential bias. Although minimal significant difference was found between patients with available specimens and those without, sample selection based on specimen availability could still introduce subtle systemic biases when sample size was small. The median duration of follow-up (68 months) was not very long for PNETs patients as many of them could survive for 5 years or more, even with recurrence. Although some bias may exist, the two independent cohorts of patients in present study and enough number of patients (more than 300) could diminish the bias as possible as we could.

In conclusion, we analyzed the proteomic profile of sporadic insulinoma and validated the expression of several proteins in a large number of PNETs. Our findings suggested that simultaneous expression of UCH-L1 and α-internexin is an independent prognostic marker for PNETs in 2 different cohorts of the patients and in the combination of both cohorts. More interestingly, the concurrent expression of both proteins is a valuable prognostic marker in a subgroup of patients with stage II and stage III tumors.

## Materials and Methods

### Clinicopathological characteristics

One hundred and seventy tumors from 164 patients with PNETs evaluated between 1989 and 2014 at Peking Union Medical College Hospital (collective I) were enrolled in the present study. Clinical follow-up data of 117 patients were obtained from 1989 until 2016. In addition, 144 tumors from 142 PNETs patients outside Peking Union Medical College Hospital (collective II) which were evaluated between 1992 and 2014 at the First Affiliated Hospital of Sun Yat-Sen University, Memorial Sloan-Kettering Cancer Center (New York), and Cedars-Sinai Medical Center (Los Angeles) were enrolled in the study, and 130 patients were followed up from 1992 until 2016 (see Supplementary Table S1). The selection of patients from each hospital was based on tumor tissue availability. All patients of the 2 cohorts underwent curative resection of primary tumors and metastasis. The duration of follow-up was calculated from the date of surgery to the date of recurrence, death, or last follow-up. Overall, 314 tumors from 306 patients and 127 paired pancreatic tissue specimens were studied. The research was approved by the Scientific Ethics Committee of Peking Union Medical College Hospital, the First Affiliated Hospital of Sun Yat-Sen University, Memorial Sloan-Kettering Cancer Center and Cedars-Sinai Medical Center, the written informed consent was obtained from all subjects, all methods were performed in accordance with the relevant guidelines and regulations. The diagnostic criteria for PNETs were identical to those used in our previous studies^29, 55, 56^. For example, the clinical and laboratory diagnostic criteria of insulinoma included symptoms of hypoglycemia, hypoglycemia (serum levels of glucose <50 mg/dl), hyperinsulinemia (elevated serum levels of insulin or high serum levels of proinsulin at time of hypoglycemia), please see our previously published paper^55^ and most recently published paper^57^. The pathological diagnosis of all PNETs was made by 2 experienced pathologists. We analyzed tumor grade and stage in 252 and 305 patients who had relevant data detail, respectively, according to ENETS guideline^42, 58^.

### Protein extraction, separation and in-gel digestion

Workflow for proteomic analysis was shown in Supplementary Fig. S3.

We deliberately selected 4 representative patients (2 female and 2 male, age 25, 40, 51, and 71) with typical insulinomas (2 G1 and 2 G2, size 1.5, 1.8, 2, and 2 cm, 2 at body/tail and 2 at head/neck of pancreas, all without lymph node or remote metastasis). Total protein was extracted from each of 4 insulinomas and their paired pancreatic tissues, and separated by electrophoresis. The protein in-gel was digested by trypsin before nanospray LC/MS/MS analysis. Please see Supplementary Method.

### Nanospray LC/MS/MS analysis and database search

The resulting peptides from 16 fractions of each sample were sequentially analyzed by nanoLC-MS/MS using an UltiMate 3000 RSLCnano System (Thermo Scientific/Dionex) coupled to Q-Exactive hybrid Quadrupole-Orbitrap Mass Spectrometer (Thermo Scientific, Bremen). The experimental procedures of mass spectrometer in detail were described in Supplementary Method.

Raw data files were searched against the NCBI/UniprotKB human protein sequence databases using the Proteome Discoverer 1.4 software (Thermo, San Jose, CA) based on the SEQUEST algorithm. The false positive discovery rates (FDR) is set on 1%.

### Spectral count based label-free quantization and statistical analysis

Protein quantification used the normalized spectral abundance factors (NSAFs) method^59, 60^ to calculate the protein relative abundance for each identified protein. In order to quantitatively describe the relative abundance, the ppm (part per million) was chosen as the unit and, the 1,000,000 ppm value was assigned to each proteome profile. A ppm value at the range of 0 to 1,000,000 ppm for each identified protein in each proteome profile was calculated based on its normalized NSAF. NSAFs were calculated as follow: NSAF_N_ = (S_N_/L_N_)/(Σ^n^_i=1_S_i_/L_i_), N is protein index, S_N_ is the number of peptide spectra matched to the protein, L_N_ is the length of protein N, N is the total number of proteins in the input database; NSAF values should range from 0 to 1, with values closer to 1 indicating higher protein levels. NSAFs were used to rank proteins within a particular analysis and to compare the relatively concentration of each identified protein across all 8 analyzed samples.

### Validating the proteins expression by immunohistochemical staining (IHC) and Western blot

Proteomic analysis showed that proteins UCH-L1, MAP1B, MAP2, VCAN, PDX-1 and CDK4 were highly expressed in all of insulinomas but not (or weakly expressed) in the paired controls. Thus, the expression of these proteins was validated by immunohistochemistry in more than 40 randomly selected PNETs. Randomization was achieved by picking every 3^rd^ patient from a patient list. The expression of CaSR was only validated in 29 insulinomas due to limitation of tissue amount. The sections of paraffin-embedded tumor tissue and paired para-tumoral tissue were antigen-retrieved by heating and stained with appropriate antibodies (see Supplementary Method for detail). The results were interpreted by 2 persons blinded to clinical data and patients’ outcome. We defined <20% tumor cells with staining of protein as negative expression, i.e. (±) and (-), similar to our previous report^29, 55^. Expression of UCH-L1, CDK4 and CaSR was further confirmed in 10 fresh frozen tissues by Western blot similar to the method previously described^29^, with anti-UCH-L1, anti-CDK4 and anti-CaSR at 1:1000, 1:800 and 1:500 dilution, respectively. Selection of the 10 fresh frozen samples (6 tumors and 4 paired tissue) was mostly based on tissue availability. The median size of the 6 tumors were 2 cm (range 1.5–4). β-actin was used as an internal control. Protein quantification was performed by reading the density of bands and, a statistic analysis (Mann-Whitney U test) on ratio of UCH-L1/β-actin was performed.

### Bioinformatic Analysis

The subcellular location of the proteins which were identified in tumors and the associated signal pathways were bioinformatically analyzed. The biological and functional features of proteins UCH-L1, MAP1B, MAP2, VCAN, PDX-1, CDK4 and α-internexin were also analyzed.

### Detecting the methylation of *UCH-L1* promoter by methylation-specific PCR (MSP) and bisulfite sequencing

Human cancer cell lines SW480 and SH-SY5Y were cultured in DMEM medium (Invitrogen, Carlsbad, CA) supplemented with 5% fetal bovine serum (HyClone, Logan, UT). Genomic DNA was isolated from the 2 cell lines, 21 fresh frozen tumoral, 9 paired tissue samples and 3 normal pancreatic tissues by ZR Genomic DNA II Kit (Zymo Research), then bisulfite-modified as we previously reported^29^. The selection of the 33 fresh frozen tumor samples was mainly based on UCH-L1 expression status as well as tissue availability. The methylation or demethylation of *UCH-L1* promoter was detected by MSP. DNA from *UCH-L1* gene unmethylated cell line SH-SY5Y and methylated cell line SW480 was used as negative and positive control, respectively. TE buffer was used as blank control of PCR. PCR conditions and primer sets were summarized in Supplementary Method. MSP results were confirmed by sequencing PCR products^29, 55^. We correlated promoter methylation status with protein expression, using statistic analysis.

### Correlating the concurrent expression of UCH-L1 and α-internexin with clinicopathological characteristics

At first, we correlated the expression of UCH-L1 protein with the clinicopathological features of 154 insulinomas and 314 PNETs, respectively. After excluding 10 patients who died of unknown reasons, the prognostic value of UCH-L1 protein was assessed in collective I by Kaplan-Meier plots. In our previous study, we found that expression of α-internexin was significantly associated with overall survival in patients with PNETs^29^. Thus, in present study, the concurrent expression of both UCH-L1 and α-internexin were correlated with clinicopathological features including prognosis in each independent collective and in the combination of 2 cohorts (n = 230). The prognostic value of UCH-L1 and α-internexin was further evaluated in 131 patients with stage II and stage III tumors. Moreover, the prognostic value of concurrent expression of UCH-L1 and α-internexin was assessed in subgroup of PNETs, i.e. insulinoma vs. non-insulinoma and NF vs functional PNETs, respectively.

### Workflow of each step of experiments

A diagram showed the number of cases used in each step of experiments, see Supplementary Table S9.

### Statistical analysis

SPSS statistics software version 20.0 was used for statistical analysis. Significance was calculated using Fisher’s exact test, χ-test for categorical variables and Mann-Whitney U test for continuous variables. Survival of patients was analyzed by Kaplan-Meier analysis and log-rank test. Cox’s proportional hazard model was used for multivariate analysis. Two-tailed test was used in all of statistical analysis. *P* ≤ 0.05 was considered significant.

## Electronic supplementary material

Supplementary Figures, Methods and Tables

Supplementary Table S2
