# Supplementary material for: Prognostic relevance of UCH-L1 and α-internexin in pancreatic neuroendocrine tumors
Source: Sci Rep. 2017 May 19;7:2205. doi: 10.1038/s41598-017-02051-1 (PMC5438410; doi:10.1038/s41598-017-02051-1)
Supplement: Supplementary file 1 — Supplementary Figures, Methods and Tables [file 41598_2017_2051_MOESM1_ESM.pdf]

Prognostic relevance of UCH-L1 and α-internexin in pancreatic neuroendocrine tumors

Yu-Li Song[1†], Run Yu [2†], Xin-Wei Qiao[1†], Chun-Mei Bai[3], Chong-Mei Lu[1], Yu Xiao[4], Ding-Rong Zhong[4], Jie Chen[4], Yu-Pei Zhao[5], Tai-Ping Zhang[5], Tian-Tian Song[1], He-Li Gao[3], Ying-Hua Wan[1], Lin Shen [6], Jie Chen[7], Bin Lv[8], Jian-Jiang Hao[9], Ye Zhang[10], Laura Tang [11], Yuan-Jia Chen[1 *]

Authors' Affiliations: [1] Department of Gastroenterology, Peking Union Medical College Hospital, Peking Union Medical College, Chinese Academy of Medical Sciences, Beijing, 100730, China, [2] Division of Endocrinology, Cedars-Sinai Medical Center, and Division of Endocrinology, Diabetes & Metabolism, UCLA, Los Angeles, California 90095, USA, [3] Department of Oncology, [4] Department of Pathology, [5] Department of Surgery, Peking Union Medical College Hospital, Peking Union Medical College, Chinese Academy of Medical Sciences, Beijing, 100730, China, [6] Department of Gastrointestinal Medical Oncology, Peking University School of Oncology, Beijing Cancer Hospital and Institute, Beijing 100142, [7] Department of Gastroenterology, the First Affiliated Hospital of Sun Yat-Sen University, Guangzhou, 510080, China, [8] Department of Gastroenterology, the First Affiliated Hospital of Zhejiang Chinese Medical University, Hangzhou, 310006, China, [9] *Poochon Scientific, Frederick*, MD 21704, USA, [10] Department of Molecular and Biochemistry, Institute of Basic Medical Sciences, Peking Union Medical College, Chinese Academy of Medical Sciences, Beijing, 100730, China, [11] Department of Pathology, Memorial Sloan-Kettering Cancer Center, New York City, 10065, USA.

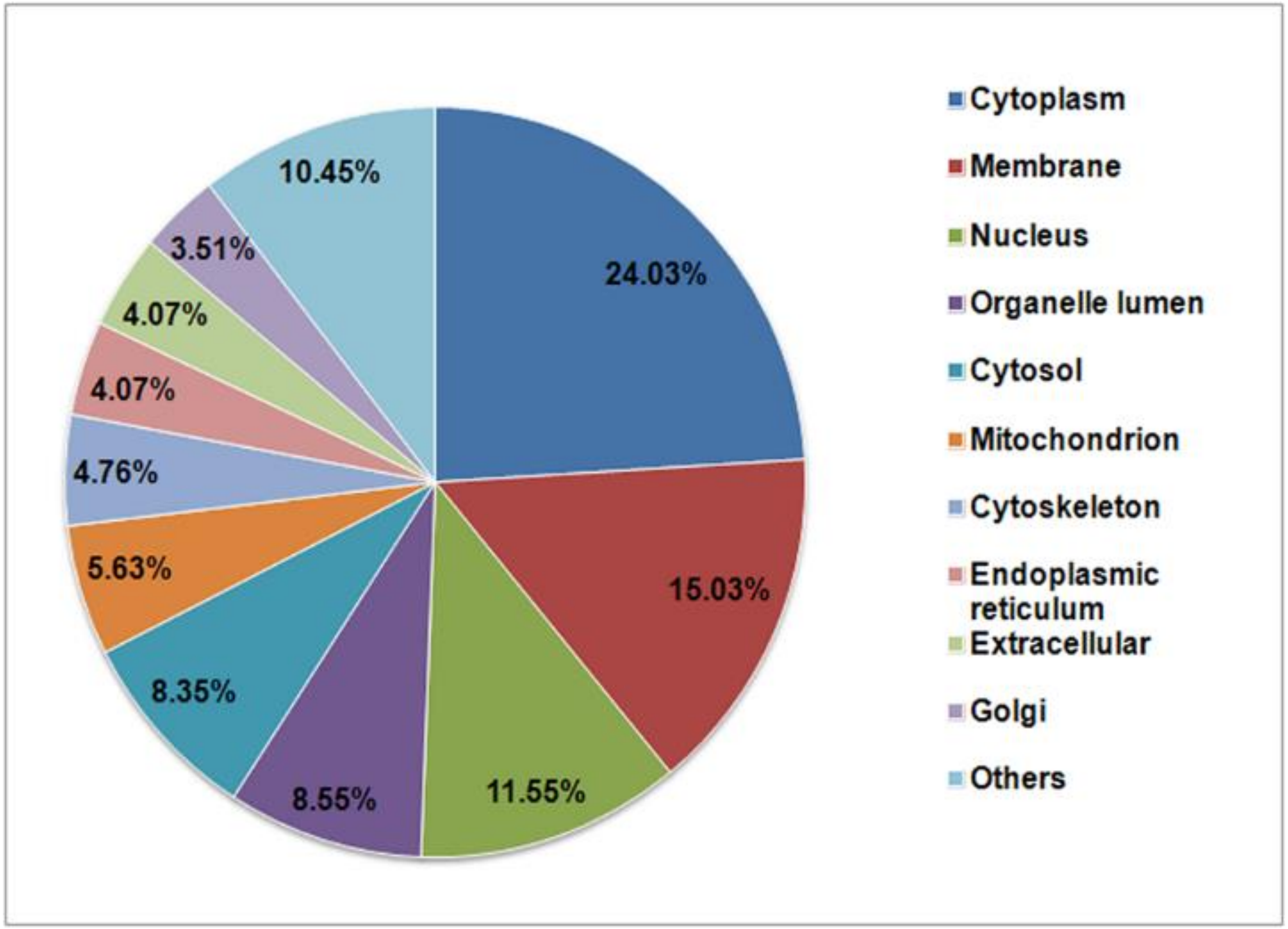

Supplementary Figure S1. Subcellular Location of Proteins Differentially Expressed in Tumors

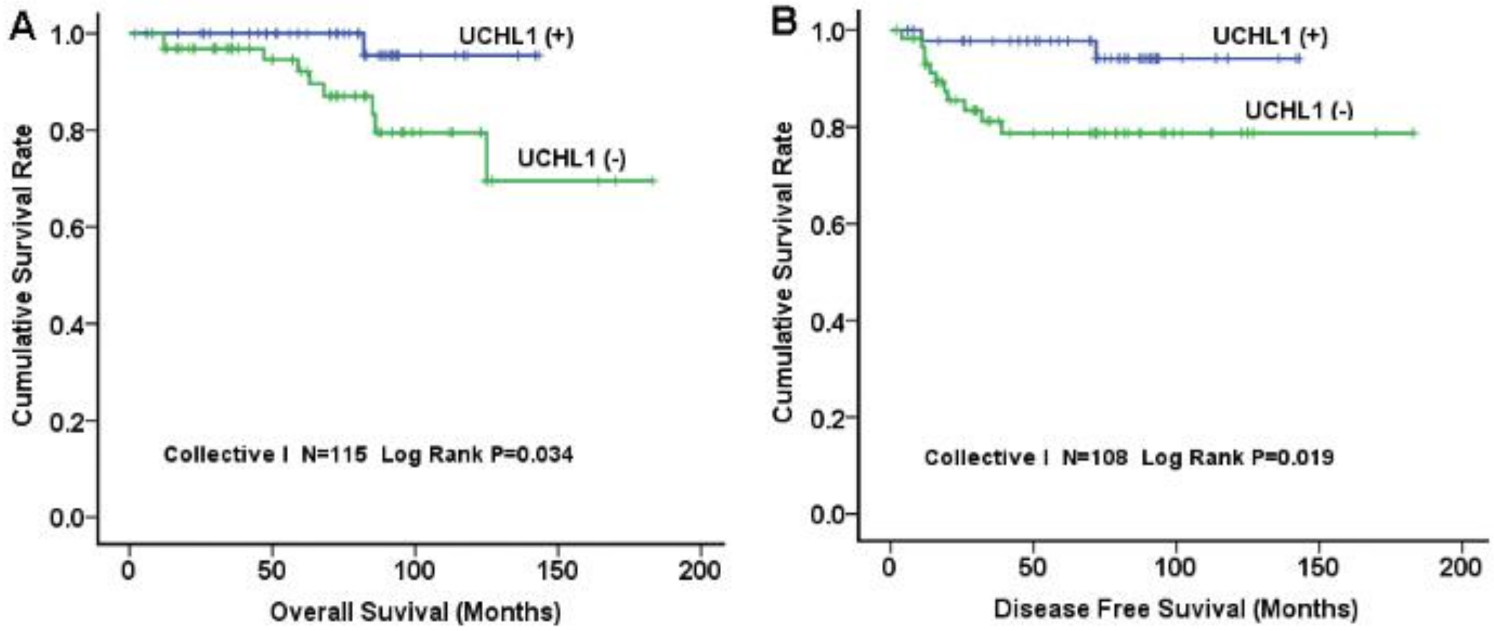

Supplementary Figure S2. The Expression of UCH-L1 and Survival in PNETs patients of collective I.

The expression of UCH-L1 in tumour significantly correlated with better overall survival (A) and disease free survival (B) in collective I.

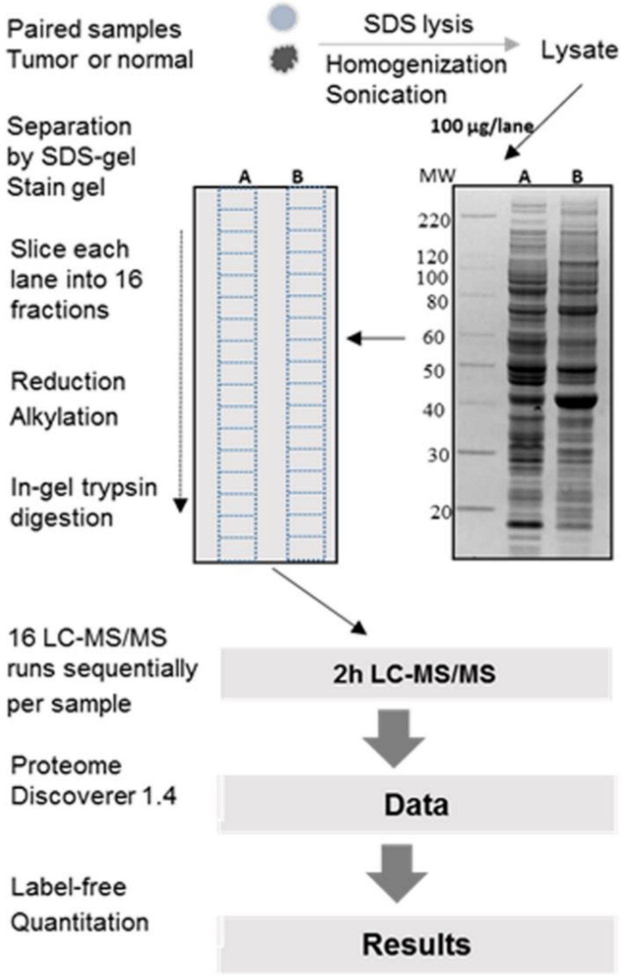

Supplementary Figure S3. Workflow for Proteomic Analysis

Supplementary Methods

Extraction, Separation and In-gel Digestion of Total Protein

Each tissue sample from 4 fresh frozen tumors and 4 paired pancreatic specimens (about 0.05 gram each sample) was ground in liquid nitrogen. The powder was lysed in 0.4 ml lyses buffer (20 mM Tris-HCl, 150 mM NaCl, 1 mM Na_2EDTA, 1 mM EGTA, 1% Triton X-100 and protease inhibitor cocktail pill, pH 7.5) and 50 µl of 10% SDS and 50 µl of 1 M DTT were added into the buffer. The mixtures were sonicated to breakdown DNA further, and then incubated at 95 ℃ for 10 min. After centrifuged at 15,000 × g for 10 min, supernatants were collected and the protein concentrations determined by BCA™ Reducing Reagent compatible assay kit (Pierce/Thermo Scientific, Rockford, USA).

Equal amounts of protein (100 µg) from each sample were loaded onto NuPAGE 4-12% Bis-Tris Gel (Life Technologies). After electrophoresis (90 min at 120V), the gel was stained with Simply Blue Safe Stain (Life Technologies), subsequently de-stained thoroughly.

For proteins digestion, the de-stained gel was washed with ion-free water 3 times, and sliced horizontally into 16 slices. Each slice was diced into tiny pieces (1–2 mm) and placed into 1.5 ml centrifuge tubes. Proteins in the gel were treated with DTT for reduction, then iodoacetamide for alkylation, and further digested by trypsin in 25 mM NH4HCO3 solution. The digested proteins were extracted and dried, then reconstituted in 0.1% formic acid before performing nanospray LC/MS/MS analysis.

Mass Spectrometer analysis

The resulting peptides from 16 fractions of each sample (4 tumor and 4 paired pancreas) were analyzed. Eight μl of each sample was loaded onto a peptide trap cartridge at a flow rate of 5 μL/min. The trapped peptides were eluted onto a reversed-phase C18 Pico Frit column (New Objective, Woburn, state) using a linear gradient of acetonitrile (3-36%) in 0.1% formic acid. The elution duration was 110 min at a flow rate of 0.3 μL/min. Eluted peptides from the PicoFrit column were ionized and sprayed into the mass spectrometer, using a Nanospray Flex Ion Source ES071 (Thermo) under the following settings: spray voltage, 1.6 kV, Capillary temperature, 250 ℃.

The Q Exactive instrument was operated in the data dependent mode to automatically switch between full scan MS and MS/MS acquisition. Survey full scan MS spectra (m/z300−2000) were acquired in the Orbitrap with 70,000 resolution (m/z 200) after accumulation of ions to a 1 × 106 target value based on predictive AGC from the previous full scan. The dynamic exclusion was set to 20 s. The 12 most intense multiply charged ions ($z \geq 2$) were sequentially isolated and fragmented by higher-energy collisional dissociation (HCD) using normalized HCD collision energy 28% with an AGC target 1e5 and a maxima injection time of 100ms at 17,500 resolution.

Antibodies and Methods for Immunohistochemical Staining

Antibody		Company / City	Catalogue number	Dilution	Antigen-retrieved method
Primary antibodies	Anti-UCH-L1	Santa Cruz Biotechnology / Santa Cruz	sc-58594	1: 50	heat retrieve
	Anti-MAP1B	Santa Cruz Biotechnology / Santa Cruz	sc-58784	1: 400	heat retrieve
	Anti-MAP2	Abnova / Taipei City	PAB3854	1:100	heat retrieve
	Anti-VCAN	Abcam / Hong kong	ab177480	1: 50	heat retrieve
	Anti-PDX-1	Abcam / Hong kong	ab47267	1: 400	heat retrieve
	Anti-CaSR	Abcam / Hong kong	ab62653	1: 50	heat retrieve
	Anti-CDK4	Santa Cruz Biotechnology / Santa Cruz	sc-23896	1: 50	heat retrieve
	Anti-α-internexin	Chemicon / Temecula	MAB5224	1:100	heat retrieve
second antibody	HRP labeled anti-mouse / rabbit IgG detection system	Zhongshan Golden Bridge Biotechnology / Beijing	PV9000	/	/

Primers and Methods for Methylation Specific PCR

The promoter of the UCHL-1 gene (Genbank accession no. NC_000004.12) was amplified by PCR focused on the -142 to +18 nucleotide (nt) region (+1 nt, transcription start site). Methylated primer and unmethylated primer were used to amplify DNA with and without methylation, respectively [49]. Touch down PCR was performed using a T1-Thermo-cycler (Biometra, Goettingen, Germany) as the following conditions.

Methylation-F: 5'-GTTCGGTCGTATTATTTCGCGT-3'; Methylation-R: 5'-CTAACGAAACCGCCCAAACT-3'

Unmethylation-F: 5'-GTTTGGTTGTATTATTTTGTGT-3'; Unmethylation-R: 5'- CTAACAAAACCACCCAAACT-3'

Methylation PCR

95℃ 15 min to activate Hotstar enzyme

(94℃ 40 sec → 61℃ 35 sec → 72℃ 35 sec) x 2 cycles

(94℃ 40 sec → 60℃ 35 sec → 72℃ 35 sec) x 2 cycles

(94℃ 40 sec → 59℃ 35 sec → 72℃ 35 sec) x 2 cycles

(94℃ 40 sec → 58℃ 35 sec → 72℃ 35 sec) x 12 cycles

(94℃ 40 sec → 56℃ 35 sec → 72℃ 35 sec) x 20 cycles

72℃ 5 min, 4℃ stop.

Unmethylation PCR

95℃ 15 min to activate Hotstar enzyme

(94℃ 35 sec → 63℃ 35 sec → 72℃ 35 sec) x 2 cycles

(94℃ 35 sec → 62℃ 35 sec → 72℃ 35 sec) x 2 cycles

(94℃ 35 sec → 61℃ 35 sec → 72℃ 35 sec) x 2 cycles

(94℃ 35 sec → 60℃ 35 sec → 72℃ 35 sec) x 30 cycles

72℃ 5 min, 4℃ stop

Supplementary Table S1 Clinicopathological Data and IHC results

Sample #	Collective	Tumor type	Gender	Age	Grade	Ki-67	Ki-67>2%	Mitosis /10 HPF	Location	T	N	M	Stage	Tumor Size	Metastasis	LN metastasis	Distal metastasis	Malignant	Recurrence	Outcome	Duration of follow up (months)	Duration of DFS (months)	INX T	INX N	UCHL1 T	UCHL1 N
1	1	Insulinoma	F	39					tail	1	0	0	1	1.5	no	no	no	no	no	DFS	114	114	+	—	+	—
2	1	Gastrinoma	F	45	2	5%	yes	1	body/tail	2	0	0	2a	2.5	no	no	no	yes	yes	AWD	83	20	—		—	
3	1	Insulinoma	F	31	2	3%	yes		head	1	0	0	1	1.6	no	no	no	no	no	DFS	89	89	—	—	+	—
4	1	Insulinoma	F	48					head	2	0	0	2a	3	no	no	no	no	no	DFS	6	6	—		+	
5	1	Insulinoma	M	15	2	5%	yes		tail	2	0	0	2a	2	no	no	no	no							—	—
6	1	Insulinoma	F	17	2	8%	yes	5.4	head	4	1	0	3b	2.5	yes	yes	no	yes	yes	DOD	12	11	+		—	
7	1	Insulinoma							lymph (metastasis)														—	—	—	—
8	1	Insulinoma							lymph (metastasis)																+	
9	1	Insulinoma	F	20	2	> 2%	yes		tail	1	0	0	1	1.3	no	no	no	no	no	DFS	56	56	+	—	+	—
10	1	Insulinoma	F	46		< 2%	no		head	1	0	0	1	1	no	no	no	no					+	—	+	—
11	1	NF	F	67	2	< 2%	no	8	head	3	0	0	2b	4.5	no	no	no	no	no	DFS	102	102	—	—	+	—
12	1	Insulinoma	M	64	1	1.7%	no	1	head	3	0	0	2b	5	no	no	no	no					—	—	+	—
13	1	Insulinoma	F	41				1	tail	1	0	0	1	1	no	no	no	no	no	DFS	93	93	+		+	
14	1	NF	F	60	1	< 2%	no	0.2	neck	2	0	0	2a	3	no	no	no	no	no	DFS	70	70	—	—	+	—
15	1	Insulinoma	M	32	1	0.1%	no	< 2	tail	1	0	0	1	1	no	no	no	no	no	DFS	48	48	—	—	+	—
16	1	NF	M	41	2	5%	yes		head	3	0	0	2b	8	no	no	no	no	no	DFS	30	30	—	—	—	—
17	1	Insulinoma	M	47		< 2%	no		body/tail	1	0	0	1	1.8	no	no	no	no					+	—	—	—
18	1	Insulinoma	F	42		< 2%	no		neck	1	0	0	1	1.5	no	no	no	no					+		+	
19	1	Insulinoma	M	39	1	0	no		head	2	0	0	2a	2	no	no	no	no	no	DFS	83	83	+		+	
20	1	Gastrinoma	F	32					liver (metastasis)	3	0	1	4	5.2	yes	no	yes	yes	no		127	127	—	—	—	—
21	1	Insulinoma	F	52					head/tail	2	0	0	2a	2	no	no	no	no	no	DFS	71	71	—	—	—	—
22	1	Insulinoma	M	67	2	> 2%	yes		body/tail	1	0	0	1	1.2	no	no	no	no	no	DFS	73	73	+		+	
23	1	NF	M	34					head/neck	2	0	0	2a	3	no	no	no	no	no		127	127	+	—	—	—
24	2	Glucagonoma	M	67	2	10%	yes		body/tail	3	0	1	4	6.1	yes	no	yes	yes	yes	AWD	73	41	—		—	—
25	1	Gastrinoma	F	52	1	< 0.5%	no	< 2	duodenum	1	1	0	3b	1.5	yes	yes	no	yes	no	DFS	79	79	—		—	—
26	1	Insulinoma	F	46	1	< 2%	no	0	head	1	0	0	1	1.5	no	no	no	no	no	DFS	95	95	+	—	—	
27	1	Insulinoma	F	40					head	2	0	0	2a	2	no	no	no	no	no	DFS	6	6	+	—	+	—
28	1	NF	F	69					head	1	0	0	1	1.5	no	no	no	no		DUC	94	9	+	—	+	—
29	1	Insulinoma	F	46	1	< 2%	no	< 2	body/tail	1	0	0	1	1.5	no	no	no	no	no	DFS	72	72	+		—	
30	1	Insulinoma	M	35	2			2	body/tail	1	0	0	1	1.5	no	no	no	no	no	DFS	8	8	+	—	+	—
31	1	NF	M	33	2	1.00%	no	3	tail	3	1	0	3b	7	yes	yes	no	yes	no	DFS	29	29	+	—	—	—
32	1	Gastrinoma	M	45	2	2.50%	yes	0	body/tail	3	0	1	4	3.5	yes	no	yes	yes	yes	AWD	36		+	—	—	—
33	1	Gastrinoma				2%	no		liver (metastasis)														+	—	—	—
34	1	NF	F	51	1	0.50%	no	0	body/tail	3	1	1	4	6	yes	yes	yes	yes	yes	DOD	86	39	—	—	—	—
35	1	NF							liver (metastasis)														—	—	—	—
36	1	Insulinoma	F	73					body	2	0	0	2a	2	no	no	no	no					+	—	+	—
37	1	NF	F	36					body/tail	1	0	1	4	1.5	yes	no	yes	yes					—		—	
38	1	Insulinoma	M	52	1	< 2%	no	1.2	head	2	0	0	2a	2.5	no	no	no	no	no	DFS	25	25	+	—	+	—
39	1	Insulinoma	M	52	1	0.10%	no	< 2	body/tail	1	0	0	1	1.3	no	no	no			survive（unknown st	48		—	—	+	—
40	1	Insulinoma	M	52	2	1%	no	2	tail	2	0	0	2a	2	no	no	no	no	no	DFS	45	45	+	—	+	
41	1	Insulinoma	F	45	1	1%	no	< 2	head	1	0	0	1	1.5	no	no	no	no					+	—	—	—
42	1	Gastrinoma	F	51	2	3%	yes	3	head/neck	3	0	1	4	4.5	yes	no	yes	yes	yes	DOD	125	12	+		—	
43	1	Insulinoma	F	49	1	< 1%	no	0.5	body/tail	2	0	0	2a	2	no	no	no	no	no	DFS	102	102	—		—	
44	1	Insulinoma	M	71	1	< 1%	no	< 2	head	2	0	0	2a	2	no	no	no	no	no	DFS	82	82	+		+	
45	1	NF	M	53					liver (metastasis)	x	0	1	4		yes	no	yes	yes	yes	DOD	47	32	-	-	—	—
46	1	Insulinoma	F	37	1	2.00%	no	0	neck	1	0	0	1	1	no	no	no	no	no	DFS	94	94	+	—	+	—
47	1	NF	M	36	1	< 2%	no	0.4	tail	2	0	0	2a	4	no	no	no	no	no	DFS	90	90	+	+	+	
48	1	Insulinoma	F	49		1%	no		neck	2	0	0	2a	2	no	no	no	no							+	—

Sample #	Collective	Tumor type	Gender	Age	Grade	Ki-67	Ki-67>2%	Mitosis /10 HPF	Location	T	N	M	Stage	Tumor Size	Metastasis	LN metastasis	Distal metastasis	Malignant	Recurrence	Outcome	Duration of follow up (months)	Duration of DFS (months)	INX T	INX N	UCHL1 T	UCHL1 N
49	1	NF	F	52					neck	2	0	0	2a	4	no	no	no	no	no	DFS	2	2	+	—	+	—
50	1	Insulinoma	M	31		< 2%	no		head	1	0	0	1	1.2	no	no	no	no					+	—	—	—
51	1	Insulinoma	M	46	2	3%	yes		tail	1	0	0	1	1.2	no	no	no	no							+	
52	1	Insulinoma	F	49	2	3%	yes	0.3	head	1	0	0	1	1.2	no	no	no	no	no	DFS	42	42	+	—	+	—
53	1	Insulinoma	F	51	1	< 1%	no	< 2	body/tail	2	0	0	2a	3	no	no	no	no	no	DFS	26	26	—	—	+	—
54	1	Insulinoma	F	46		< 1%	no		head	1	0	0	1	1	no	no	no	no							+	
55	1	Insulinoma	F	59	2	> 2%	yes		neck	2	0	0	2a	2.9	no	no	no	no	no	DFS	87	87	+	—	—	—
56	1	Insulinoma	M	53	1	< 2%	no	< 2	body/tail	1	0	0	1	1.5	no	no	no	no	no	DFS	82	82	+		—	
57	1	Insulinoma	F	24					body/tail	2	0	0	2a	2	no	no	no	no							+	—
58	1	NF	F	42					head	3	0	0	2b	5	no	no	no	no					+		—	
59	1	Insulinoma	M	41	1	1%	no	< 2	body/tail	1	0	0	1	1	no	no	no	no	no	DFS	23	23	+		—	—
60	1	NF	F	29					head	2	0	0	2a	3	no	no	no	no					—		—	—
61	1	Insulinoma	M	46					head	2	0	0	2a	3	no	no	no	no							+	—
62	1	Insulinoma	F	44		< 2%	no		head	2	0	0	2a	2	no	no	no	no	no	DFS	83	83	+	—	—	—
63	1	Insulinoma	M	52					head	1	0	0	1	1	no	no	no	no					+	—	+	—
64	1	NF	M	72	2	> 2%	yes		body	1	0	0	1	1.7	no	no	no	no	no	DFS	123	123	+	—	—	—
65	1	Insulinoma	M	48		< 2%	no		head	2	0	0	2a	2	no	no	no	no	no	DFS	80	80	+	—	+	—
66	1	Insulinoma	M	30	2	< 1%	no	4	body	2	0	0	2a	2	no	no	no	no	no	DFS	94	94	+	—	+	—
67	1	Insulinoma	F	51	2	> 2%	yes		tail	1	0	0	1	1.8	no	no	no	no	no	DFS	48	48	+	—	+	—
68	1	Insulinoma	M	50	2	8%	yes	3.5	head	2	0	1	4	3	yes	no	yes	yes	yes	DOD	12		—	—	—	—
69	1	Insulinoma	M	48					head	2	0	0	2a	3	no	no	no	no							+	—
70	1	Gastrinoma	M	43					head	1	1	1	4	1.5	yes	yes	yes	yes							—	
71	1	Glucagonoma	F	51	2	8%	yes	0	body/tail	3	0	1	4	8	yes	no	yes	yes	yes	DOD	85	12	+		—	
72	1	Insulinoma	F	31	2	> 2%	yes		head	2	0	0	2a	2.2	no	no	no	no	no	DFS	51	51	+	—	+	—
73	1	Insulinoma	F	27					body	1	0	0	1	1.5	no	no	no	no							+	—
74	1	Insulinoma	M	53	1	1%	no	<2	head	2	0	0	2a	2.5	no	no	no	no	no	DFS	30	30	—		—	
75	1	Insulinoma	F	60					head	1	0	0	1	1.8	no	no	no	no	no	DFS	18	18	+		—	
76	1	Insulinoma	F	51	1	<1%	no	<2	head	1	0	0	1	1.3	no	no	no	no	no	DFS	12	12	+		—	—
77	1	VIPoma							liver (metastasis)					8									—	—	+	—
78	1	VIPoma	M	41	2	3%	yes	0	body/tail	2	1	1	4	3.5	yes	yes	yes	yes	yes	AWD	**92**	26	—	—	—	—
79	1	Insulinoma	M	58	1	< 2%	no	0.4	head	2	0	0	2a	3	no	no	no	no	no	DFS	123	123	—		—	
80	1	NF	F	33	2	10%	yes	0	body/tail	2	0	1	4	2.3	yes	no	yes	yes	yes	AWD	22	16	—	—	—	—
81	1	Insulinoma	M	66	2	> 2%			body/tail	4	0	1	4	5	yes	no	yes	yes					—		—	—
82	1	Insulinoma	M	58	1	< 2%	no	<2	tail	1	0	0	1	1.5	no	no	no	no	no	DFS	93	93	+	—	+	—
83	1	Insulinoma	F	55	1	1%	no	0	head	1	0	0	1	1	no	no	no	no	no	DFS	96	96	+		—	
84	1	Insulinoma	M	36	1	< 2%	no	0	tail	2	0	0	2a	2	no	no	no	no	no	DFS	28	28	+	—	+	—
85	1	Insulinoma	M	38	1	1%	no	<2	head	1	0	0	1	1.6	no	no	no	no					+	—	—	—
86	1	NF	F	57					body/tail	2	0	0	2a	4	no	no	no	no					+	—	+	—
87	1	Insulinoma	M	39		< 2%	no		neck	1	0	0	1	1	no	no	no	no					+	—	+	—
88	1	Insulinoma	F	71	1	< 2%	no	<2	body/tail	1	0	0	1	1	no	no	no	no	no	DFS	99	99	—	—	—	—
89	1	Insulinoma	F	42	2	> 2%	yes		tail	1	0	0	1	1.5	no	no	no	no	no	DFS	91	91	+	—	+	—
90	1	Gastrinoma	M	39	2	9%	yes	4.5	head/neck	2	0	1	4	3.5	yes	no	yes	yes	yes	AWD	164	4	+	—	—	
91	1	Insulinoma	M	29	1	1.1%	no	<2	body/tail	2	0	0	2a	2.2	no	no	no	no	no	DFS	92	92	+	—	+	—
92	1	Insulinoma	F	71	1	1.5%	no	<2	head	1	0	0	1	1.2	no	no	no	no	no	DFS	72	72	+	—	+	—
93	1	Insulinoma	F	46	1	0.10%	no	1	neck	1	0	0	1	1.2	no	no	no	no	no	DFS	88	88	+	—	+	—
94	1	Insulinoma	F	43	1	1.2%	no	<2	body/tail	1	0	0	1	1.1	no	no	no	no	no	DFS	73	73	+	—	—	—
95	1	NF	F	46	2	7%	yes		body/tail	2	0	0	2a	2	no	no	no	no	no	DFS	59	59	+	—	+	—
96	1	Insulinoma	F	63	1	0.20%	no	<2	body/tail	1	0	0	1	1.5	no	no	no			survive (unknown st	50		—	—	—	
97	1	Insulinoma	F	36					head	2	0	1	4	4	yes	no	yes	yes					+		+	
98	1	Gastrinoma	F	37	2	2.60%	yes	0.5	body/tail	2	1	1	4	4	yes	yes	yes	yes	yes	DOD	63	19	—	—	—	

Sample #	Collective	Tumor type	Gender	Age	Grade	Ki-67	Ki-67>2%	Mitosis /10 HPF	Location	T	N	M	Stage	Tumor Size	Metastasis	LN metastasis	Distal metastasis	Malignant	Recurrence	Outcome	Duration of follow up (months)	Duration of DFS (months)	INX T	INX N	UCHL1 T	UCHL1 N
99	1	Insulinoma	F	26	1	0.10%	no	0	head/body/tail	1	0	0	1	1.5	no	no	no	no	no	DFS	92	92	+	-	+	—
100	1	Insulinoma	M	66	1	0.10%	no	1.2	body/tail	2	0	0	2a	2	no	no	no	no	no	DFS	38	38	—	—	—	
101	1	Insulinoma	M	41	1	<2%	no	0.2	head	2	0	0	2a	2	no	no	no	no					+	—	+	—
102	1	Gastrinoma	F	69	2	0	no	3.3	stomach	3	1	0	3b	4.5	yes	yes	no	yes	no	DFS	70	70	—	—	—	
103	1	Insulinoma	M	42	1	0.10%	no	<2	head	2	0	0	2a	2.5	no	no	no	no	no	DFS	96	96	—	—	—	
104	1	Insulinoma	F	20	2	> 2%	yes		tail	2	0	0	2a	3.5	no	no	no	no	no	DFS	87	87	+	—	+	—
105	1	Insulinoma	M	37	2	3.5%	yes		body/tail	1	0	0	1	1.8	no	no	no	no	no	DFS	16	16	+	—	—	—
106	1	NF	F	40	1	1%	no	0.7	head	2	0	0	2a	2.5	no	no	no	no	no	DFS	75	75	—	—	—	—
107	1	Insulinoma	F	74	1	1%	no	< 2	body/tail	1	0	0	1	1.5	no	no	no	no	no	DFS	70	70	+	—	+	—
108	1	Insulinoma	F	42	1	< 2%	no	< 2	body/tail	1	0	0	1	1.3	no	no	no	no	no	DFS	170	170	—	—	—	—
109	1	Insulinoma	F	59	1	1%	no	< 2	head	1	0	0	1	1.5	no	no	no	no					+	—	—	—
110	1	Insulinoma	F	33	2	< 1%	no	6	body	3	1	0	3b	2.2	yes	yes	no	yes	no	DFS	52	52	+	—	+	—
111	1	Insulinoma	F	56	1	< 2%	no	<2	body/tail	1	0	0	1	1.5	no	no	no	no	no	DFS	83	83	+		+	
112	1	Insulinoma	F	40	1	< 1%	no	<2	head	1	0	0	1	1.5	no	no	no	no	no	DFS	62	62	+	—	+	—
113	1	NF	M	37	1	1.50%	no	1.5	body/tail	3	0	1	4	14	yes	no	yes	yes	yes	AWD	60	14	—		—	
114	1	NF	F	30	1	0	no	0.7	tail	3	0	0	2b	6	no	no	no	no	no	DFS	113	113	—	—	—	—
115	1	Insulinoma	M	62	1	1.50%	no	<2	body/tail	2	0	0	2a	2	no	no	no	no	no	DFS	17	17	—		+	
116	1	Insulinoma	F	66	1	< 2%	no	0	head	2	0	0	2a	2	no	no	no	no	no	DFS	21	21	+	—	—	—
117	1	Insulinoma	F	24	2	5%	yes		head	1	0	0	1	1.2	no	no	no	no					+	—	—	—
118	1	Glucagonoma	F	45	1	0	no	0.5	body/tail	2	0	1	4	2.5	yes	no	yes	yes		DOD	68		—		—	
119	1	Insulinoma	F	25	1	1.50%	no	<2	head	1	0	0	1	1.5	no	no	no	no	no	DFS	75	75	+		+	
120	1	Glucagonoma	F	51	2	4.50%	yes	1.25	body/tail	3	0	1	4	6	yes	no	yes	yes	yes	AWD	117	72	—		+	
121	1	NF	F	29		< 2%	no		head	2	0	0	2a	4	no	no	no	no					—	—	+	—
122	2	NF	F	33	2	5%	yes		body/tail	4	0	0	3a	5.5	no	no	no	yes	yes	AWD	16	71	—		+	
123	**2**	NF	F	17	1	< 2%	no	0		3	0	0	2b	9	no	no	no	no					—	—	—	—
124	2	NF	M	33	1	< 1%	no	<2		3	1	1	4	6	yes	yes	yes	yes					—		—	
125	1	Glucagonoma	F	45	1	1.20%	no	1	body/tail	3	0	1	4	4.5	yes	yes	yes	yes	yes	DOD	82	11	—	—	+	—
126	2	NF	F	64					tail	3	0	0	2b	11	no	no	no	no					—	—	—	—
127	2	Insulinoma	M	85	2	10%	yes	5	body	3	0	1	4	6.1	yes	no	yes	yes	yes	DOD	46	35	—	—	+	—
128	2	NF	F	47	1	0.5%	no	1	neck	3	0	0	2b	5	no	no	no	no	no	DFS	60	60	—	—	—	—
129	2	NF	F	84	1	< 2%	no	0	head	3	0	0	2b	4.3	no	no	no	no		DUC	12	12	+		—	
130	2	NF	F	55	2			5	tail	3	0	0	2b	10	no	no	no	no	no	DFS	133	133	+		+	
131	2	NF	F	65	1	< 2%	no	0	head	3	0	0	2b	5.2	no	no	no	no		DUC	101	101	—		—	
132	2	NF	F	58	1	< 2%	no	0	tail	1	0	0	1	1.2	no	no	no	no	no	DFS	140	140	+		+	
133	2	NF	F	44	2			5	head	2	1	0	3b	3.2	yes	yes	no	yes	yes	DOD	105	34	—		+	—
134	2	NF	M	61	2			5	head	2	0	0	2a	4	no	no	no	no	no	DFS	13	13	+		—	
135	2	NF	F	63	1	< 2%	no	0	tail	2	0	0	2a	3	no	no	no	no	no	DFS	123	123	—		—	
136	2	NF	F	48	1	< 2%	no	0	body/tail	3	1	0	3b	4.4	yes	yes	no	yes	no	DFS	118	118	—		—	
137	2	NF	M	45	2			7	head	3	0	0	2b	4.5	no	no	no	yes	yes	DOD	72	69	—		—	
138	2	NF	M	42	1	< 2%	no	1	head	2	0	0	2a	3.1	no	no	no	yes	yes	DOD	87	31	+		—	
139	2	NF	F	80	1	< 2%	no	0	head	2	0	0	2a	2.5	no	no	no	no	no	DFS	106	106	—		+	
140	2	VIPoma	F	64	1	< 2%	no	0	head	3	0	0	2b	4.5	no	no	no	no	no	DFS	137	137	—		—	
141	2	NF	F	75	1	< 2%	no	0	body	2	0	0	2a	3.3	no	no	no	no	no	DFS	36	36	—		—	
142	2	NF	F	74	1	< 2%	no	0	head	2	0	0	2a	3.5	no	no	no	no	no	DFS	110	110	+		—	
143	2	NF	F	47	1	< 2%	no	1	head	3	1	0	3b	4.2	yes	yes	no	yes	yes	AWD	133	21	+		—	
144	2	NF	F	47	2			6	tail	2	0	1	4	2.5	yes	no	yes	yes	yes	DOD	126	8	—		++	
145	2	NF	F	38	1	< 2%	no	0	tail	3	0	0	2b	7.2	no	no	no	no	no	DFS	12	12	—		++	
146	2	NF	F	47					tail	3	0	0	2b	5	no	no	no	no	no	DFS	56	56	+		++	
147	2	NF	M	41	1	< 2%	no	0	body/tail	3	0	1	4	7	yes	no	yes	yes	yes	AWD	58	28	+		—	
148	2	NF	M	74	2			3	tail	2	0	0	2a	3.4	no	no	no	no	no	DFS	64	64	—		—	

Sample #	Collective	Tumor type	Gender	Age	Grade	Ki-67	Ki-67>2%	Mitosis /10 HPF	Location	T	N	M	Stage	Tumor Size	Metastasis	LN metastasis	Distal metastasis	Malignant	Recurrence	Outcome	Duration of follow up (months)	Duration of DFS (months)	INX T	INX N	UCHL1 T	UCHL1 N
149	2	Gastrinoma	F	30	2	< 2%	no	2	head	3	1	0	3b	6.5	yes	yes	no	yes	yes	AWD	113	14	+		+	
150	2	Insulinoma	M	75	1	< 2%	no	0	body	1	0	0	1	1.5	no	no	no	no	no	DFS	34	34	+		—	
151	2	NF	F	71	1	< 2%	no	1	tail	3	0	0	2b	7.8	no	no	no	no	no	DFS	5	5	+		—	
152	2	Insulinoma	F	58	2			12	body	2	0	0	2a	4	no	no	no	no	no	DFS	4	4	+		++	
153	2	NF	M	66	1	< 2%	no	1	tail	3	1	0	3b	7.9	yes	yes	no	yes	yes	AWD	103	96	—		—	
154	2	NF	M	60	2			6	tail	3	0	0	2b	5.5	no	no	no	no	no	DFS	66	66	—		—	
155	2	Glucagonoma	M	47	2			2	tail	2	0	0	2a	3.5	no	no	no	yes	yes	AWD	44	14	—		—	
156	2	NF	M	44					body	3	1	0	3b	12	yes	yes	no	yes	no	DFS	81	81	+		+	
157	2	NF	M	56	2	5%		14	body/tail	3	1	1	4	4.2	yes	yes	yes	yes	yes	DOD	37		—		—	
158	2	NF	M	59	1	< 2%	no	1	tail	3	0	0	2b	4.3	no	no	no	no	no	DFS	25	25	+		—	
159	2	NF	M	26	2			6	tail	2	0	0	2a	3	no	no	no	no	no	DFS	34	34	+		—	
160	2	NF	M	79	2			3	head	2	0	0	2a	2.8	no	no	no	no	no	DFS	75	75	+		++	
161	2	NF	M	57	1	< 2%	no	0	head	1	0	0	1	1.4	no	no	no	no	no	DFS	54	54	—		—	
162	2	NF	M	52	1	< 2%	no	0	body	1	0	0	1	1.1	no	no	no	no	no	DFS	28	28	+		—	
163	2	NF	M	47	1	< 1%	no	0	body/tail	1	0	0	1	1	no	no	no	no	no	DFS	36	36	—		—	
164	2	NF	F	45	2			10	head	3	1	0	3b	10.5	yes	yes	no	yes	yes	DOD	67	6	—		+	
165	2	NF	M	70	2			7	tail	3	0	0	2b	4.5	no	no	no	no	no	DFS	47	47	—		+	
166	2	NF	M	48	1	< 2%	no	0	head	2	0	0	2a	2.5	no	no	no	no	no	DFS	8	8	+		—	
167	2	NF	F	57	1	< 2%	no	1	body/tail	2	0	0	2a	4	no	no	no	no	no	DFS	95	95	+		—	
168	2	NF	M	52	2			3	head	3	1	1	4	4.4	yes	yes	yes	yes	yes	DOD	90	45	+		+	
169	2	NF	M	68	1	< 2%	no	1	body	2	0	0	2a	2.5	no	no	no	no	no	DFS	100	1	—		—	
170	2	NF	F	55	1	< 2%	no	0	head	2	0	0	2a	2.5	no	no	no	no	no	DFS	34	34	+		+	
171	2	NF	F	64	1	< 2%	no	0	head	3	0	0	2b	4.5	no	no	no	no	no	DFS	59	59	—		—	
172	2	NF	F	40	1	< 2%	no	0	tail	3	0	0	2b	5.2	no	no	no	no	no	DFS	82	82	—		—	
173	2	NF	F	68	1	< 2%	no	1	tail	2	0	0	2a	3	no	no	no	no	no	DUC	76	76	—		—	
174	2	Insulinoma	M	53	2			6	tail	2	0	0	2a	4.2	no	no	no	yes	yes	AWD	45	14	+		—	
175	2	NF	F	37	2	5%	yes	3	head	3	1	1	4	7.5	yes	yes	yes	yes	yes	DOD	24	2	—		—	
176	2	NF	M	59	1	< 2%	no	0	tail	2	0	0	2a	2.6	no	no	no	no	no	DFS	74	74	+		—	
177	2	NF	M	55	2			2	tail	3	1	0	3b	11	yes	yes	no	yes	yes	AWD	98	15	—		—	
178	2	NF	F	43	2	5%	yes	1	head	3	0	0	2b	17	no	no	no	yes	yes	AWD	85	34	+		—	
179	2	NF	F	68	2	3%	yes	1	head	2	1	0	3b	2	yes	yes	no	yes	no	DFS	85	85	—		—	
180	2	NF	M	62	2	5%	yes	12	body/tail	2	0	0	2a	3.1	no	no	no	no	no	DFS	37	37	+		+	
181	2	NF	M	63	2	3%	yes	5	body/tail	2	0	0	2a	4	no	no	no	no	no	DFS	20	20	—		—	
182	2	NF	M	62	1	< 2%	no	0	tail	2	0	0	2a	3.6	no	no	no	no	no	DFS	49	49	+		—	
183	2	NF	M	65					tail	3	1	0	3b	5	yes	yes	no	yes	no	DFS	42	42	—		—	
184	2	NF	M	47					tail	3	0	1	4	14.5	yes	no	yes	yes	yes	AWD	31		—		—	
185	2	NF	F	52	2	2%	no	9	tail	3	1	0	3b	6.2	yes	yes	no	yes	yes	AWD	74	1	—		+	
186	2	NF	M	71	2	> 2%	yes	1	tail	3	1	0	3b	5.7	yes	yes	no	yes	no	DFS	72	72	—		+	
187	2	NF	F	35	2	< 2%	no	14	head	3	1	1	4	4.5	yes	yes	yes	yes	yes	DOD	62		+		—	
188	2	NF	F	47					body/tail	x	0	1	4		yes	no	yes	yes	yes	DOD	218	72	—		—	
189	2	Insulinoma	M	38	1	< 2%	no	0	body	1	0	0	1	1.3	no	no	no	no		DUC	11	11	+		+	
190	2	NF	F	51	2			18	body/tail	3	0	1	4	15	yes	no	yes	yes	yes	DOD	96	57	—		+	
191	2	NF	F	40	1			2	head	3	1	1	4	4	yes	yes	yes	yes	yes	DOD	36		—		—	
192	2	Insulinoma	F	52	1	< 2%	no	1	body/tail	2	0	0	2a	4	no	no	no	no	no	DFS	24	24	+		—	
193	2	Gastrinoma	F	65	1	< 2%	no	0	head	1	1	0	3b	1.2	yes	yes	no	yes		DUC	4	4	—		—	
194	2	NF	M	71	1	< 2%	no	0	body/tail	1	0	0	1	1.8	no	no	no	no	no	DFS	131	131	+		—	
195	2	NF	M	70	2			20	body/tail	3	0	0	2b	8	no	no	no	yes	yes	DOD	40	18	—		—	
196	2	NF	M	61	2			12	body/tail	2	0	0	2a	3.4	no	no	no	no		DUC	15	15	—		—	
197	2	NF	F	43	2			2	tail	1	0	0	1	1.8	no	no	no	no	no	DFS	154	154	—		—	
198	2	NF	F	71	2			2	body/tail	1	0	0	1	1.5	no	no	no	no	no	DFS	154	154	—		+	

Sample #	Collective	Tumor type	Gender	Age	Grade	Ki-67	Ki-67>2%	Mitosis /10 HPF	Location	T	N	M	Stage	Tumor Size	Metastasis	LN metastasis	Distal metastasis	Malignant	Recurrence	Outcome	Duration of follow up (months)	Duration of DFS (months)	INX T	INX N	UCHL1 T	UCHL1 N
199	2	NF	F	50	1	< 2%	no	1	head	2	1	0	3b	2.5	yes	yes	no	yes	no	DFS	37	37	—		+	
200	2	NF	F	70	1	< 2%	no	1	body/tail	3	0	0	2b	6.5	no	no	no	no		DUC	131	131	—		+	
201	2	VIPoma	F	65					body/tail	x	x	1	4		yes		yes	yes	yes	DOD	151	35	—		+	
202	2	NF	M	46	1	< 2%	no	0	head	2	0	0	2a	2.2	no	no	no	no	no	DFS	99	99	+		+	
203	2	NF	M	41	2			2	body/tail	2	0	0	2a	3	no	no	no	no	no	DFS	56	56	—		—	
204	2	NF	M	75	2			17	body/tail	2	1	0	3b	3	yes	yes	no	yes	yes	DOD	39	4	—		—	
205	2	NF	F	53	2			4	tail	3	0	0	2b	15	no	no	no	no	no	DFS	136	136	+		+	
206	2	Insulinoma	F	75	1	< 2%	no	1	tail	1	0	0	1	1	no	no	no	no	no	DFS	95	95			+	
207	2	NF	M	48	2			2	head	3	0	0	2b	5.8	no	no	no	no	no	DFS	1	1			—	
208	2	NF	F	46	2			3	tail	2	0	0	2a	3	no	no	no	no	no	DFS	117	117	+		—	
209	2	NF	F	42	1	<1%	no	0	tail	2	0	0	2a	2	no	no	no	no	no	DFS	119	119			—	
210	2	NF	M	70	2	4%	yes	1	tail	1	0	0	1	1.8	no	no	no	no	no	DFS	77	77			—	
211	2	NF	M	63	1	< 2%	no	0	tail	1	0	0	1	1.8	no	no	no	no	no	DFS	89	89			—	
212	2	NF	F	46					tail	3	0	0	2b	10	no	no	no	no	no	DFS	1	1			—	
213	2	NF	F	54	1	< 2%	no	0	tail	3	0	0	2b	8.5	no	no	no	no	no	DFS	7	7			+	
214	2	NF	F	54	2			3	head	3	1	0	3b	8.4	yes	yes	no	yes	yes	DOD	35	15	—		+	
215	2	NF	M	68	2			14	head	2	0	0	2a	2.1	no	no	no	no		DUC	62	62			—	
216	2	NF	F	46					tail	3	1	1	4	7.5	yes	yes	yes	yes							—	
217	2	NF	M	47					head	3	0	0	2b	6	no	no	no	no	no	DFS	60	60	+		+	
218	2	Insulinoma	M	58	1	< 1%	no	0	tail	3	x	1	4	7	yes		yes	yes					—		+	—
219	2	Insulinoma	F	27	2	< 1%	no	4	body/tail	1	0	0	1	1.8	no	no	no	no					+		+	—
220	2	Glucagonoma	F	63	1	< 1%	no	<1	body	2	0	0	2a	2	no	no	no	no	no	DFS	190	190	—	—	+	—
221	2	Insulinoma	M	19	1	< 1%	no	0	head/body/tail	2	0	0	2a	2	no	no	no	no	no	DFS	110	110	+	—	+	—
222	2	NF	M	34	1	< 1%	no		head	3	0	0	2b	8	no	no	no	no	no	DFS	154	154	—		—	
223	2	NF	F	61	1	< 1%	no	0	head	3	1	0	3b	8	yes	yes	no	yes					—		+	—
224	2	NF	M	35	2	0	no	6		3	x	1	4	8	yes		yes	yes		DOD	2		—		—	
225	2	NF	M	54	1	< 1%	no	0	tail	3	0	0	2b	3	no	no	no	yes		DOD	5		—		—	
226	2	NF	M	62	1	< 1%	no	<2	head	3	x	1	4	3	yes		yes	yes					—		+	—
227	2	NF	F	48	1	< 1%	no	0	body	2	0	0	2a	2	no	no	no	no	no	DOD	103	63	—	—	+	—
228	2	NF	F	23	1	< 1%	no		head	2	x	1	4	4	yes		yes	yes					—		—	
229	2	Insulinoma	M	23	2	< 1%	no	6	head	1	0	0	1	1.5	no	no	no	no					+		+	
230	2	Insulinoma	F	53	1	< 1%	no	0	head	1	0	0	1	1.5	no	no	no	no	no	DFS	104	104	+	—	+	—
231	2	Insulinoma	F	54	1	< 1%	no	0.6	head	2	0	0	2a	2	no	no	no	no	no	DFS	92	92	+	—	+	—
232	2	Insulinoma	F	51	1	< 1%	no	0.4	body/tail	2	0	0	2a	3	no	no	no	no	no	DFS	41	41	—		+	
233	2	Insulinoma	M	56	1	< 1%	no		tail	2	0	0	2a	3	no	no	no	no	no	DFS	87	87	—	—	—	—
234	2	Insulinoma	F	53	1	2%	no		head	1	0	0	1	1.5	no	no	no	no	no	DFS	24	24	—	—	+	
235	2	Insulinoma	F	39	2	10%	yes	7	tail	2	0	0	2a	2.5	no	no	no	no	no	DFS	83	83	+	—	+	—
236	2	Insulinoma	M	59	2	5%	yes		tail	2	0	0	2a	2	no	no	no	no	no	DFS	82	82	+	—	+	—
237	2	Insulinoma	F	62	1	< 1%	no	1	neck	1	0	0	1	1.5	no	no	no	no	no	DFS	22	22	—		+	
238	2	Insulinoma	M	57	2	< 1%	no	3	tail	2	0	0	2a	2	no	no	no	no	no	DFS	81	81	—		+	—
239	2	Insulinoma	F	46	1	< 1%	no	0	body/tail	1	0	0	1	1.5	no	no	no	no	no	DFS	80	80	—	—	+	—
240	2	Insulinoma	M	30	1	< 1%	no		neck	2	0	0	2a	2	no	no	no	no	no	DFS	69	69	+		+	
241	2	Insulinoma	F	30	1	< 1%	no	<2	tail	1	0	0	1	1.5	no	no	no	no	no	DFS	57	57	+	—	+	—
242	2	NF	F	57	3	> 20%	yes		body	4	1	1	4	4	yes	yes	yes	yes	no	DOD	37	15	—	—	+	—
243	2	NF	M	56	1	2%	no		body/tail	4	0	1	4	12	no	no	yes	yes	no	DFS	65	65	+	—	—	—
244	2	NF	F	39	1	1%	no		head	3	0	0	2b	9	no	no	no	no	no	DFS	60	60	+		+	
245	2	NF	F	40	2	3%	yes		head	3	1	0	3b	4	yes	yes	no	yes	yes	DOD	16	11	—		+	
246	2	NF	F	68	3	60%	yes		head/neck	2	0	0	2a	2	no	no	no	yes	yes	DOD	26	1	—		—	
247	2	NF	M	39	2	5%	yes		head	3	0	0	2b	7	no	no	no	yes	no	DFS	56	56	+		—	
248	2	NF	M	59	3	30%	yes	20	body/tail	4	x	1	4	5	yes		yes	yes	yes	DOD	19		+	—	—	—

Sample #	Collective	Tumor type	Gender	Age	Grade	Ki-67	Ki-67>2%	Mitosis /10 HPF	Location	T	N	M	Stage	Tumor Size	Metastasis	LN metastasis	Distal metastasis	Malignant	Recurrence	Outcome	Duration of follow up (months)	Duration of DFS (months)	INX T	INX N	UCHL1 T	UCHL1 N
249	2	NF	M	32	3	30%	yes	2	head	4	0	0	3a	6	no	no	no	yes	yes	DOD	33	12	—		—	
250	1	Insulinoma	F	46					body/tail	1	0	0	1	1	no	no	no	no					+	—	+	
251	1	Insulinoma	F	43						1	0	0			no	no	no	no							—	
252	1	Insulinoma	M	22		< 2%	no		body/tail	1	0	0	1	1	no	no	no	no					+	—	+	—
253	1	Insulinoma	F	56	1	1%	no		body/tail	2	0	0	2a	2	no	no	no	no	no	DFS	88	88	+		—	
254	1	Insulinoma	M	54	2	> 2%	yes		head/neck	2	0	0	2a	2.5	no	no	no	no	no	DFS	118	118	+	—	+	—
255	1	Insulinoma	F	74	1	1%	no	0.2	body/tail	2	0	0	2a	3	no	no	no	no	no	DFS	79	79	+		—	
256	1	Insulinoma	F	69	2	20%	yes	3	body/tail	1	0	0	1	1.5	no	no	no	no	no	DFS	35	35	+	—	—	—
257	1	Insulinoma	F	18	2	> 2%	yes	0.7	body/tail	1	0	0	1	0.7	no	no	no	no	no	DFS	42	42	—	—	+	—
258	1	Insulinoma	M	16	2	10%	yes		body/tail	1	0	0	1	1.5	no	no	no	no	no	DFS	30	30	+	—	—	
259	1	Insulinoma	F	57	1	< 1%	no	1	body/tail	1	0	0	1	1	no	no	no	no					+	—	+	—
260	1	Insulinoma	F	36	1	0.10%	no	0.5	head/neck	2	0	0	2a	2	no	no	no	no					+	—	—	
261	1	Insulinoma	M	52	1	< 1%	no	1	body/tail	1	0	0	1	1.2	no	no	no	no	no	DFS	57	57	+	—	—	—
262	1	Insulinoma	F	58		1%	no		body/tail	2	0	0	2a	2	no	no	no	no							—	—
263	1	Insulinoma	M	42	2	2%	yes		head/neck	1	0	0	1	1.5	no	no	no	no	no	DFS	72	72	+	—	—	
264	1	Insulinoma	F	68				1	body/tail	2	0	0	2a	2.7	no	no	no	no	no	DFS	62	62	+	—	—	
265	1	Insulinoma	F	66		1%	no		body/tail	2	0	0	2a	2	no	no	no	no							—	—
266	1	Insulinoma	M	47	1	< 2%	no	0.3	head/neck	1	0	0	1	1	no	no	no	no	no	DFS	17	17	+	—	—	—
267	1	Insulinoma	F	34					body/tail	1	0	0	1	0.8	no	no	no	no							—	
268	1	Insulinoma	F	19	2	2%	no	3	head/neck	1	0	0	1	1.2	no	no	no	no	no	DFS	13	13	+	—	—	
269	1	Insulinoma	F	32	1	0.10%	no	1	head/neck	2	0	0	2a	2	no	no	no	no	no	DFS	8	8	+	—	—	
270	1	Insulinoma	M	40					head/neck	1	0	0	1	1.2	no	no	no	no							+	
271	1	Insulinoma	F	40		<1%	no		head/neck	1	0	0	1	1.6	no	no	no	no					+	—	—	—
272	2	Glucagonoma	M	70	2	5%	yes		body/tail	3	1	0	3b	8	yes	yes	yes	yes	yes	AWD	53		—		—	—
273	2	Gastrinoma	M	47	1	2%	no		head	2	0	0	2a	2.5	no	no	no	no	no	DFS	90	90	—		—	
274	2	Glucagonoma	F	54	3	45%	yes		head	3	1	1	4	4.2	yes	yes	yes	yes		AWD	2		+	—	—	—
275	2	Insulinoma	F	54	2	< 1%	no	4	body/tail	3	1	1	4	4.8	yes	yes	yes	yes	yes	DOD	19	12	—		—	
276	1	Insulinoma	F	44	1	< 2%	no		body/tail	1	0	0	1	1.2	no	no	no	no	no	DFS	96	96	—		—	
277	1	Insulinoma	F	32	2	0.10%	no	3	body/tail	1	0	0	1	1.5	no	no	no	no	no	DFS	142	142	+	—	+	—
278	1	Insulinoma	M	51	1	< 2%	no	0.5	body/tail	2	0	1	4	4	yes	no	yes	yes	yes	DOD	59		—	—	—	—
279	1	Insulinoma							liver (metastasis)					1.6									—		—	
280	1	Insulinoma	F	56	1	0.10%	no	1	Tail	1	0	0	1	1.8	no	no	no	no	no	DFS	143	143	—	—	+	—
281	1	Insulinoma	F	36	1			0	head	2	0	0	2a	2	no	no	no	no	no	DFS	136	136	+	—	+	—
282	1	Insulinoma	F	41	2	<0.1%	no	2	head	1	0	0	1	1.2	no	no	no	no	no	DFS	125	125	+	—	—	—
283	1	Insulinoma	M	51	1	< 2%	no	1	head	2	0	0	2a	2	no	no	no	yes	yes	AWD	36		—	—	—	—
284	1	Insulinoma	F	23	1	0.20%	no	0	head	1	0	0	1	1.5	no	no	no	no	no	DFS	80	80	+	—	+	
285	1	Insulinoma	M	44	1	< 2%	no		head	1	0	0	1	1	no	no	no	no	no	DFS	77	77	+		+	
286	1	Insulinoma	F	37	1	1%	no	1	Tail	1	0	0	1	0.8	no	no	no	no	no	DFS	112	112	+	—	—	—
287	2	NF	M	31	2	1%	no	7	head	4	1	1	4	1.7	yes	yes	yes	yes	no	DOD	9	6	—	—	+	—
288	2	NF							scalp (metastasisr)														—		+	
289	1	NF	F	22	1	< 1%	no		body/tail	2	0	0	2a	4	no	no	no	no	no	DFS	36	36	+		+	
290	1	Insulinoma	F	40	1	2%	no	< 2	head	1	0	0	1	1.5	no	no	no	no					+	—	+	
291	1	Insulinoma	F	64	2	3%	yes		body/tail	2	0	0	2a	2	no	no	no	no	no	DFS	125	125	+	—	—	—
292	1	Insulinoma	F	46					Tail	1	0	0	1	1.2	no	no	no	no					+	—	—	—
293	2	NF	F	46	2	3%	yes		head	2	1	0	3b	3.5	yes	yes	no	yes	yes	DOD	23	12	—		+	—
294	2	NF							lymph (metastasis)														—		—	
295	1	Insulinoma	M	32	2	5%	yes			2	0	0	2a	2	no	no	no	no	no	DFS	42	42	—	—	—	—
296	1	Insulinoma	M	43	1	0	no		head	1	0	0	1	1.5	no	no	no						+		—	
297	2	NF	F	61	1	2%	no		neck	3	0	0	2b	7	no	no	no	yes	yes	AWD	59	33	—		—	
298	2	NF	F	79	2	3%	yes		body/tail	3	0	0	2b	5.5	no	no	no	yes	yes	AWD	85	49	+	-	—	—

Sample #	Collective	Tumor type	Gender	Age	Grade	Ki-67	Ki-67>2%	Mitosis /10 HPF	Location	T	N	M	Stage	Tumor Size	Metastasis	LN metastasis	Distal metastasis	Malignant	Recurrence	Outcome	Duration of follow up (months)	Duration of DFS (months)	INX T	INX N	UCHL1 T	UCHL1 N
299	2	NF	F	52	2	10%	yes	3	body/tail	2	1	0	3b	3.5	yes	yes	no	yes	yes	AWD	69	63	+		+	
300	2	NF	M	49	2	5%	yes		body/tail	2	1	0	3b	3.3	yes	yes	no	yes	yes	AWD	58	24	+		—	
301	2	NF	M	61	2	10%	yes		body/tail	3	0	1	4	8	yes	no	yes	yes	yes				+		+	
302	2	NF	F	46	2	15%	yes	11	tail	3	0	1	4	4.5	yes	no	yes	yes	yes				—	—	+	—
303	2	NF	M	60	3	60%	yes	> 20	tail	4	0	0	3a	9	no	no	no	yes	yes	DFS	17	10	+	—	—	
304	2	NF	M	55	2	20%	yes	10	tail	2	0	0	2a	2.5	no	no	no	no	no	DFS	38	38	—	—	—	—
305	1	Insulinoma	F	43	1	1%	no	< 2	head/neck	1	0	0	1	1	no	no	no	no	no	DFS	2	2	—	—	—	—
306	1	Insulinoma	M	61	1	1%	no	0	body/tail	2	0	0	2a	2	no	no	no	no	no	DFS	183	183	—	—	—	
307	1	Glucagonoma	M	34	1	0	no		head/body/tail	2	0	0	2a	3.9	no	no	no	no					+	—	+	—
308	1	Insulinoma	F	44	2	4%	yes		head/neck	1	0	0	1	1.5	no	no	no	no	no	DFS	34	34	+		—	
309	1	Insulinoma	M	67	1	1.6%	no	< 2	head	1	0	0	1	1.1	no	no	no	no	no	DUC	36		—	—	—	
310	1	Insulinoma	M	66	1	1%	no	< 2	head/neck	1	0	0	1	1	no	no	no	no					+	-	+	—
311	1	Insulinoma	F	29	1	1.5%	no	< 2	head/neck	2	0	0	2a	2	no	no	no	no					+	-	—	—
312	1	Insulinoma	M	67					tail	1	0	0	1	1.5	no	no	no	no	no	DFS	50	50	+		—	
313	1	Insulinoma	F	58	1	1%	no	< 2	head/neck	1	0	0	1	1.5	no	no	no	no					—	—	—	—
314	1	Insulinoma	M	37	1	1.5%	no	< 2	body/tail	2	0	0	2a	3	no	no	no	no					—		—	

Supplementary Table S3 Signal pathways

Ingenuity Canonical Pathways	-log(p-value)	Ratio	Downregulated	No change	Upregulated	No overlap with dataset	Molecules
G Beta Gamma Signaling	8.21	0.10	0/121 (0%)	0/121 (0%)	12/121 (10%)	109/121 (90%)	PRKACB,GNAS,PRKAR2B,MAPK1,GRB2,MAPK3,GNAO1,GNAI1,PRKACA,GNAQ,HRAS,GNAZ
IL-1 Signaling	6.97	0.10	0/109 (0%)	0/109 (0%)	11/109 (10%)	98/109 (90%)	PRKACB,ECSIT,GNAS,MAPK14,PRKAR2B,MAPK1,GNAO1,GNAI1,PRKACA,GNAQ,GNAZ
Acute Phase Response Signaling	6.73	0.08	0/181 (0%)	0/181 (0%)	14/181 (8%)	167/181 (92%)	ECSIT,HPX,FN1,APOH,MAPK1,GRB2,AHSG,SERPINF1,HRAS,PLG,MAPK14,ITIH2,C4BPA,MAPK3
GNRH Signaling	6.35	0.08	1/153 (1%)	0/153 (0%)	11/153 (7%)	141/153 (92%)	PRKACB,GNAS,MAPK14,PRKAR2B,MAPK1,GRB2,MAPK3,GNAI1,PRKACA,GNAQ,PLCB3,HRAS
Melatonin Signaling	6.11	0.11	1/81 (1%)	0/81 (0%)	8/81 (10%)	72/81 (89%)	PRKACB,PRKAR2B,MAPK1,MAPK3,GNAO1,GNAI1,PRKACA,GNAQ,PLCB3
Cardiac Hypertrophy Signaling	5.98	0.06	2/249 (1%)	0/249 (0%)	13/249 (5%)	234/249 (94%)	PRKACB,GNAS,MAPK1,GRB2,GNAQ,GNAI1,HRAS,GNAZ,MAPK14, PRKAR2B,MAPK3,GNAO1,EIF2B5,PRKACA,PLCB3
CREB Signaling in Neurons	5.75	0.06	1/207 (0%)	0/207 (0%)	12/207 (6%)	194/207 (94%)	PRKACB,GNAS,MAPK1,GRB2,GNAI1,GNAQ,HRAS,GNAZ,PRKAR2B,MAPK3,GNAO1,PRKACA,PLCB3
Parkinson's Signaling	5.62	0.26	0/19 (0%)	0/19 (0%)	5/19 (26%)	14/19 (74%)	UCHL1,SEPT5,MAPK14,MAPK1,CYCS
Gap Junction Signaling	5.51	0.07	1/181 (1%)	0/181 (0%)	11/181 (6%)	169/181 (93%)	PRKACB,DBN1,GNAS,PRKAR2B,MAPK1,GRB2,MAPK3,GNAI1,PRKACA,GNAQ,PLCB3,HRAS
AMPK Signaling	5.37	0.06	1/180 (1%)	0/180 (0%)	10/180 (6%)	169/180 (94%)	PRKACB,GNAS,MAPK14,PRKAR2B,MAPK1,PRKACA,PPM1A,CFTR,PFKP,PFKFB2,PFKM
Renin-Angiotensin Signaling	5.36	0.08	0/126 (0%)	0/126 (0%)	10/126 (8%)	116/126 (92%)	PRKACB,GNAS,MAPK14,PRKAR2B,MAPK1,GRB2,MAPK3,PRKACA,GNAQ,HRAS
β-Adrenergic Signaling	5.27	0.08	0/109 (0%)	0/109 (0%)	9/109 (8%)	100/109 (92%)	PRKACB,GNAS,PRKAR2B,MAPK1,MAPK3,GNAI1,PRKACA,GNAQ,HRAS
Androgen Signaling	5.26	0.07	0/145 (0%)	0/145 (0%)	10/145 (7%)	135/145 (93%)	PRKACB,GNAS,PRKAR2B,MAPK1,MAPK3,GNAO1,GNAI1,PRKACA,GNAQ,GNAZ
Corticotropin Releasing Hormone Signaling	5.22	0.07	0/145 (0%)	0/145 (0%)	10/145 (7%)	135/145 (93%)	PRKACB,GNAS,MAPK14,PRKAR2B,MAPK1,MAPK3,GNAO1,GNAI1,PRKACA,GNAQ
UDP-N-acetyl-D-galactosamine Biosynthesis II	5.06	0.16	1/25 (4%)	0/25 (0%)	3/25 (12%)	21/25 (84%)	HK1,GPI,GNPDA2,UAP1
Ephrin Receptor Signaling	5.00	0.06	0/210 (0%)	0/210 (0%)	12/210 (6%)	198/210 (94%)	ACTR2,GNAS,ARPC1A,MAPK1,GRB2,MAPK3,GNAO1,GNAI1,GNAQ,HRAS,GNAZ,RASA1
P2Y Purigenic Receptor Signaling Pathway	4.93	0.07	1/144 (1%)	0/144 (0%)	9/144 (6%)	134/144 (93%)	PRKACB,GNAS,PRKAR2B,MAPK1,MAPK3,GNAI1,PRKACA,GNAQ,PLCB3,HRAS
Ephrin B Signaling	4.92	0.10	0/82 (0%)	0/82 (0%)	8/82 (10%)	74/82 (90%)	GNAS,MAPK1,MAPK3,GNAO1,GNAI1,GNAQ,HRAS,GNAZ
Leptin Signaling in Obesity	4.88	0.09	1/85 (1%)	0/85 (0%)	7/85 (8%)	77/85 (91%)	PRKACB,GNAS,PRKAR2B,MAPK1,GRB2,MAPK3,PRKACA,PLCB3
BMP signaling pathway	4.88	0.09	0/85 (0%)	0/85 (0%)	8/85 (9%)	77/85 (91%)	PRKACB,MAPK14,PRKAR2B,MAPK1,GRB2,MAPK3,PRKACA,HRAS
Role of NFAT in Cardiac Hypertrophy	4.82	0.06	1/209 (0%)	0/209 (0%)	11/209 (5%)	197/209 (94%)	PRKACB,GNAS,MAPK14,PRKAR2B,MAPK1,GRB2,MAPK3,GNAI1,PRKACA,GNAQ,PLCB3,HRAS
Actin Cytoskeleton Signaling	4.80	0.05	2/242 (1%)	0/242 (0%)	11/242 (5%)	229/242 (95%)	KNG1,MYH10,ACTR2,FN1,MAPK1,GRB2,HRAS,GSN,IQGAP2,DIAPH1,ARPC1A,MAPK3,PFN2
Axonal Guidance Signaling	4.74	0.04	1/483 (0%)	0/483 (0%)	18/483 (4%)	464/483 (96%)	DPYSL2,PRKACB,ACTR2,GNAS,MAPK1,GRB2,GNAQ,GNAI1,HRAS,GNAZ, PRKAR2B,ARPC1A,MAPK3,GNAO1,PRKACA,PLCB3,PFN2,PSMD14,RASA1
Neuroprotective Role of THOP1 in Alzheimer's Disease	4.64	0.11	0/55 (0%)	0/55 (0%)	6/55 (11%)	49/55 (89%)	PRKACB,KNG1,PLG,PRKAR2B,HLA-A,PRKACA
Relaxin Signaling	4.49	0.06	0/164 (0%)	0/164 (0%)	10/164 (6%)	154/164 (94%)	PRKACB,GNAS,PRKAR2B,MAPK1,MAPK3,GNAO1,GNAI1,PRKACA,GNAQ,GNAZ
Melanocyte Development and Pigmentation Signaling	4.44	0.08	0/95 (0%)	0/95 (0%)	8/95 (8%)	87/95 (92%)	PRKACB,GNAS,PRKAR2B,MAPK1,GRB2,MAPK3,PRKACA,HRAS
Molecular Mechanisms of Cancer	4.37	0.04	1/387 (0%)	0/387 (0%)	15/387 (4%)	371/387 (96%)	PRKACB,GNAS,MAPK1,GRB2,GNAQ,GNAI1,HRAS,GNAZ,MAPK14,PRKAR2B, MAPK3,GNAO1,PRKACA,PLCB3,CYCS,RASA1
Endothelin-1 Signaling	4.37	0.06	1/191 (1%)	0/191 (0%)	10/191 (5%)	180/191 (94%)	GNAS,MAPK14,MAPK1,GRB2,MAPK3,GNAO1,GNAI1,GNAQ,PLCB3,HRAS,GNAZ
Chemokine Signaling	4.19	0.09	1/75 (1%)	0/75 (0%)	6/75 (8%)	68/75 (91%)	MAPK14,MAPK1,MAPK3,GNAI1,GNAQ,PLCB3,HRAS
NRF2-mediated Oxidative Stress Response	4.17	0.06	3/195 (2%)	0/195 (0%)	8/195 (4%)	184/195 (94%)	MGST1,MAPK14,MAPK1,MAPK3,DNAJA4,GCLC,HRAS,AOX1,DNAJB6,DNAJA1,TXNRD1
IGF-1 Signaling	4.07	0.07	0/107 (0%)	0/107 (0%)	8/107 (7%)	99/107 (93%)	PRKACB,PRKAR2B,MAPK1,GRB2,MAPK3,PRKACA,HRAS,RASA1
Amyloid Processing	4.03	0.10	0/61 (0%)	0/61 (0%)	6/61 (10%)	55/61 (90%)	PRKACB,MAPK14,PRKAR2B,MAPK1,MAPK3,PRKACA
Systemic Lupus Erythematosus Signaling	3.96	0.05	0/256 (0%)	0/256 (0%)	12/256 (5%)	244/256 (95%)	KNG1,PRPF19,LSM12,SNRPB,MAPK1,GRB2,HLA-A,MAPK3,HRAS,C8B,C8G,SNRNP40
G-Protein Coupled Receptor Signaling	3.96	0.05	1/276 (0%)	0/276 (0%)	12/276 (4%)	263/276 (95%)	PRKACB,PRKAR2B,GNAS,MAPK1,GRB2,MAPK3,GNAO1,PRKACA,GNAQ,GNAI1,PLCB3,HRAS,RASA1
Thrombin Signaling	3.94	0.05	1/211 (0%)	0/211 (0%)	10/211 (5%)	200/211 (95%)	GNAS,MAPK14,MAPK1,GRB2,MAPK3,GNAO1,GNAI1,GNAQ,PLCB3,HRAS,GNAZ
Breast Cancer Regulation by Stathmin1	3.90	0.05	1/214 (0%)	0/214 (0%)	10/214 (5%)	203/214 (95%)	PRKACB,GNAS,PRKAR2B,MAPK1,GRB2,MAPK3,GNAI1,PRKACA,GNAQ,PLCB3,HRAS
EGF Signaling	3.80	0.09	0/64 (0%)	0/64 (0%)	6/64 (9%)	58/64 (91%)	MAPK14,MAPK1,GRB2,MAPK3,HRAS,RASA1
Role of NFAT in Regulation of the Immune Response	3.68	0.05	1/200 (1%)	0/200 (0%)	9/200 (5%)	190/200 (95%)	GNAS,MAPK1,GRB2,MAPK3,GNAO1,GNAI1,GNAQ,PLCB3,HRAS,GNAZ
Synaptic Long Term Depression	3.67	0.06	1/161 (1%)	0/161 (0%)	8/161 (5%)	152/161 (94%)	GNAS,MAPK1,MAPK3,GNAO1,GNAI1,GNAQ,PLCB3,HRAS,GNAZ
CDK5 Signaling	3.53	0.07	0/97 (0%)	0/97 (0%)	7/97 (7%)	90/97 (93%)	PRKACB,GNAS,PRKAR2B,MAPK1,MAPK3,PRKACA,HRAS
Glycolysis I	3.43	0.10	0/41 (0%)	0/41 (0%)	4/41 (10%)	37/41 (90%)	GPI,PFKP,PFKM,ALDOC
Synaptic Long Term Potentiation	3.43	0.06	1/130 (1%)	0/130 (0%)	7/130 (5%)	122/130 (94%)	PRKACB,PRKAR2B,MAPK1,MAPK3,PRKACA,GNAQ,PLCB3,HRAS
LXR/RXR Activation	3.40	0.06	0/139 (0%)	0/139 (0%)	8/139 (6%)	131/139 (94%)	KNG1,HPX,APOH,VTN,AHSG,SERPINF1,A1BG,HADH
CXCR4 Signaling	3.38	0.05	1/174 (1%)	0/174 (0%)	8/174 (5%)	165/174 (95%)	GNAS,MAPK1,MAPK3,GNAO1,GNAI1,GNAQ,PLCB3,HRAS,GNAZ
Signaling by Rho Family GTPases	3.24	0.04	0/262 (0%)	0/262 (0%)	11/262 (4%)	251/262 (96%)	ACTR2,SEPT5,GNAS,ARPC1A,MAPK1,MAPK3,GNAO1,GNAI1,GNAQ,GNAZ,SEPT6
Glutaryl-CoA Degradation	3.21	0.13	1/24 (4%)	0/24 (0%)	2/24 (8%)	21/24 (88%)	ACAT2,EHHADH,HADH
Cholecystokinin/Gastrin-mediated Signaling	3.16	0.07	1/106 (1%)	0/106 (0%)	6/106 (6%)	99/106 (93%)	MAPK14,MAPK1,GRB2,MAPK3,GNAQ,PLCB3,HRAS
Insulin Receptor Signaling	3.15	0.05	1/149 (1%)	0/149 (0%)	7/149 (5%)	141/149 (95%)	PRKACB,PRKAR2B,MAPK1,GRB2,MAPK3,EIF2B5,PRKACA,HRAS
Semaphorin Signaling in Neurons	3.03	0.09	0/54 (0%)	0/54 (0%)	5/54 (9%)	49/54 (91%)	DPYSL2,CRMP1,MAPK1,DPYSL3,MAPK3
Sphingosine-1-phosphate Signaling	2.94	0.06	1/123 (1%)	0/123 (0%)	6/123 (5%)	116/123 (94%)	GNAS,MAPK1,MAPK3,GNAI1,GNAQ,PLCB3,ASAH1
Complement System	2.89	0.11	0/35 (0%)	0/35 (0%)	4/35 (11%)	31/35 (89%)	C4BPA,C8B,C1QC,C8G
Oncostatin M Signaling	2.84	0.11	0/35 (0%)	0/35 (0%)	4/35 (11%)	31/35 (89%)	MAPK1,GRB2,MAPK3,HRAS
CCR3 Signaling in Eosinophils	2.83	0.05	1/134 (1%)	0/134 (0%)	6/134 (4%)	127/134 (95%)	GNAS,MAPK14,MAPK1,MAPK3,GNAI1,PLCB3,HRAS
Glioma Invasiveness Signaling	2.81	0.08	0/66 (0%)	0/66 (0%)	5/66 (8%)	61/66 (92%)	PLG,MAPK1,MAPK3,VTN,HRAS
p70S6K Signaling	2.72	0.05	1/132 (1%)	0/132 (0%)	6/132 (5%)	125/132 (95%)	MAPK1,GRB2,MAPK3,GNAI1,GNAQ,PLCB3,HRAS

Ingenuity Canonical Pathways	-log(p-value)	Ratio	Downregulated	No change	Upregulated	No overlap with dataset	Molecules
Acetate Conversion to Acetyl-CoA	2.69	0.18	1/11 (9%)	0/11 (0%)	1/11 (9%)	9/11 (82%)	ACSS1,ACSL1
UVA-Induced MAPK Signaling	2.67	0.06	1/98 (1%)	0/98 (0%)	5/98 (5%)	92/98 (94%)	MAPK14,MAPK1,MAPK3,PLCB3,HRAS,CYCS
Cdc42 Signaling	2.60	0.04	2/186 (1%)	0/186 (0%)	6/186 (3%)	178/186 (96%)	ACTR2,IQGAP2,DIAPH1,MAPK14,ARPC1A,MAPK1,HLA-A,RASA1
Antioxidant Action of Vitamin C	2.55	0.05	1/110 (1%)	0/110 (0%)	5/110 (5%)	104/110 (95%)	MAPK14,MAPK1,MAPK3,PLCB3,NXN,TXNRD1
JAK/Stat Signaling	2.53	0.07	0/71 (0%)	0/71 (0%)	5/71 (7%)	66/71 (93%)	MAPK1,GRB2,MAPK3,GNAQ,HRAS
Thioredoxin Pathway	2.52	0.25	0/8 (0%)	0/8 (0%)	2/8 (25%)	6/8 (75%)	NXN,TXNRD1
eNOS Signaling	2.51	0.05	1/155 (1%)	0/155 (0%)	6/155 (4%)	148/155 (95%)	PRKACB,KNG1,GNAS,PRKAR2B,PRKACA,GNAQ,AQP1
Agrin Interactions at Neuromuscular Junction	2.51	0.07	2/70 (3%)	0/70 (0%)	3/70 (4%)	65/70 (93%)	MAPK1,MAPK3,LAMA2,UTRN,HRAS
GDNF Family Ligand-Receptor Interactions	2.51	0.07	0/76 (0%)	0/76 (0%)	5/76 (7%)	71/76 (93%)	MAPK1,GRB2,MAPK3,HRAS,RASA1
UVC-Induced MAPK Signaling	2.50	0.09	0/44 (0%)	0/44 (0%)	4/44 (9%)	40/44 (91%)	MAPK14,MAPK1,MAPK3,HRAS
Tryptophan Degradation III (Eukaryotic)	2.48	0.06	1/48 (2%)	0/48 (0%)	2/48 (4%)	45/48 (94%)	ACAT2,EHHADH,HADH
Neuropathic Pain Signaling In Dorsal Horn Neurons	2.46	0.06	1/109 (1%)	0/109 (0%)	5/109 (5%)	103/109 (94%)	PRKACB,PRKAR2B,MAPK1,MAPK3,PRKACA,PLCB3
Nitric Oxide Signaling in the Cardiovascular System	2.44	0.05	0/125 (0%)	0/125 (0%)	6/125 (5%)	119/125 (95%)	PRKACB,KNG1,PRKAR2B,MAPK1,MAPK3,PRKACA
PEDF Signaling	2.43	0.06	0/79 (0%)	0/79 (0%)	5/79 (6%)	74/79 (94%)	MAPK14,MAPK1,MAPK3,SERPINF1,HRAS
Rac Signaling	2.40	0.05	1/127 (1%)	0/127 (0%)	5/127 (4%)	121/127 (95%)	ACTR2,IQGAP2,ARPC1A,MAPK1,MAPK3,HRAS
FLT3 Signaling in Hematopoietic Progenitor Cells	2.32	0.06	0/79 (0%)	0/79 (0%)	5/79 (6%)	74/79 (94%)	MAPK14,MAPK1,GRB2,MAPK3,HRAS
IL-22 Signaling	2.31	0.12	0/25 (0%)	0/25 (0%)	3/25 (12%)	22/25 (88%)	MAPK14,MAPK1,MAPK3
Gluconeogenesis I	2.31	0.06	0/48 (0%)	0/48 (0%)	3/48 (6%)	45/48 (94%)	GPI,ME1,ALDOC
Dopamine Receptor Signaling	2.30	0.05	0/96 (0%)	0/96 (0%)	5/96 (5%)	91/96 (95%)	PRKACB,GNAS,PRKAR2B,PRKACA,QDPR
PDGF Signaling	2.27	0.06	0/86 (0%)	0/86 (0%)	5/86 (6%)	81/86 (94%)	MAPK1,GRB2,MAPK3,HRAS,RASA1
IL-17A Signaling in Gastric Cells	2.26	0.11	0/28 (0%)	0/28 (0%)	3/28 (11%)	25/28 (89%)	MAPK14,MAPK1,MAPK3
Role of JAK family kinases in IL-6-type Cytokine Signaling	2.26	0.11	0/28 (0%)	0/28 (0%)	3/28 (11%)	25/28 (89%)	MAPK14,MAPK1,MAPK3
Triacylglycerol Degradation	2.26	0.09	2/32 (6%)	0/32 (0%)	1/32 (3%)	29/32 (91%)	ABHD12,CEL,PNLIPRP1
CNTF Signaling	2.16	0.07	0/57 (0%)	0/57 (0%)	4/57 (7%)	53/57 (93%)	MAPK1,GRB2,MAPK3,HRAS
Endometrial Cancer Signaling	2.16	0.07	0/60 (0%)	0/60 (0%)	4/60 (7%)	56/60 (93%)	MAPK1,GRB2,MAPK3,HRAS
TGF-β Signaling	2.14	0.06	0/90 (0%)	0/90 (0%)	5/90 (6%)	85/90 (94%)	MAPK14,MAPK1,GRB2,MAPK3,HRAS
IL-2 Signaling	2.13	0.07	0/61 (0%)	0/61 (0%)	4/61 (7%)	57/61 (93%)	MAPK1,GRB2,MAPK3,HRAS
Sperm Motility	2.12	0.04	2/143 (1%)	0/143 (0%)	4/143 (3%)	137/143 (96%)	PRKACB,SLC16A10,GNAS,PRKAR2B,PRKACA,PLCB3
ErbB Signaling	2.10	0.06	0/90 (0%)	0/90 (0%)	5/90 (6%)	85/90 (94%)	MAPK14,MAPK1,GRB2,MAPK3,HRAS
Thrombopoietin Signaling	2.08	0.06	0/64 (0%)	0/64 (0%)	4/64 (6%)	60/64 (94%)	MAPK1,GRB2,MAPK3,HRAS
FGF Signaling	2.07	0.05	0/94 (0%)	0/94 (0%)	5/94 (5%)	89/94 (95%)	MAPK14,MAPK1,GRB2,MAPK3,HRAS
Ketolysis	2.06	0.11	0/19 (0%)	0/19 (0%)	2/19 (11%)	17/19 (89%)	ACAT2,OXCT1
Actin Nucleation by ARP-WASP Complex	2.05	0.06	0/67 (0%)	0/67 (0%)	4/67 (6%)	63/67 (94%)	ACTR2,ARPC1A,GRB2,HRAS
Superpathway of Cholesterol Biosynthesis	2.03	0.03	0/87 (0%)	0/87 (0%)	3/87 (3%)	84/87 (97%)	NSDHL,ACAT2,IDI1
Sonic Hedgehog Signaling	2.03	0.09	0/35 (0%)	0/35 (0%)	3/35 (9%)	32/35 (91%)	PRKACB,PRKAR2B,PRKACA
ErbB2-ErbB3 Signaling	2.02	0.06	0/63 (0%)	0/63 (0%)	4/63 (6%)	59/63 (94%)	MAPK1,GRB2,MAPK3,HRAS
Regulation of Cellular Mechanics by Calpain Protease	2.02	0.05	0/73 (0%)	0/73 (0%)	4/73 (5%)	69/73 (95%)	MAPK1,GRB2,MAPK3,HRAS
Dopamine-DARPP32 Feedback in cAMP Signaling	2.00	0.04	1/187 (1%)	0/187 (0%)	6/187 (3%)	180/187 (96%)	PRKACB,GNAS,PRKAR2B,GNAI1,PRKACA,GNAQ,PLCB3
4-1BB Signaling in T Lymphocytes	1.99	0.08	0/36 (0%)	0/36 (0%)	3/36 (8%)	33/36 (92%)	MAPK14,MAPK1,MAPK3
Estrogen Receptor Signaling	1.97	0.04	1/136 (1%)	0/136 (0%)	5/136 (4%)	130/136 (96%)	PCK2,MAPK1,GRB2,MAPK3,HRAS,CARM1
VEGF Signaling	1.95	0.05	1/109 (1%)	0/109 (0%)	4/109 (4%)	104/109 (95%)	MAPK1,GRB2,MAPK3,EIF2B5,HRAS
ErbB4 Signaling	1.95	0.06	0/69 (0%)	0/69 (0%)	4/69 (6%)	65/69 (94%)	MAPK1,GRB2,MAPK3,HRAS
Cellular Effects of Sildenafil (Viagra)	1.94	0.04	1/155 (1%)	0/155 (0%)	5/155 (3%)	149/155 (96%)	PRKACB,MYH10,GNAS,PRKAR2B,PRKACA,PLCB3
IL-15 Signaling	1.92	0.06	0/72 (0%)	0/72 (0%)	4/72 (6%)	68/72 (94%)	MAPK14,MAPK1,MAPK3,HRAS
Mouse Embryonic Stem Cell Pluripotency	1.90	0.05	0/99 (0%)	0/99 (0%)	5/99 (5%)	94/99 (95%)	MAPK14,MAPK1,GRB2,MAPK3,HRAS
G Protein Signaling Mediated by Tubby	1.88	0.07	1/44 (2%)	0/44 (0%)	2/44 (5%)	41/44 (93%)	GNAS,GNAQ,PLCB3
Ovarian Cancer Signaling	1.88	0.04	0/152 (0%)	0/152 (0%)	6/152 (4%)	146/152 (96%)	PRKACB,PRKAR2B,MAPK1,MAPK3,PRKACA,HRAS
GM-CSF Signaling	1.87	0.06	0/68 (0%)	0/68 (0%)	4/68 (6%)	64/68 (94%)	MAPK1,GRB2,MAPK3,HRAS
RhoGDI Signaling	1.87	0.03	0/201 (0%)	0/201 (0%)	7/201 (3%)	194/201 (97%)	ACTR2,GNAS,ARPC1A,GNAO1,GNAI1,GNAQ,GNAZ
T Cell Receptor Signaling	1.86	0.05	0/109 (0%)	0/109 (0%)	5/109 (5%)	104/109 (95%)	MAPK1,GRB2,MAPK3,HRAS,RASA1
RAR Activation	1.86	0.04	0/191 (0%)	0/191 (0%)	7/191 (4%)	184/191 (96%)	PRKACB,GNAS,MAPK14,PRKAR2B,MAPK1,PRKACA,CARM1
IL-17A Signaling in Fibroblasts	1.85	0.08	0/40 (0%)	0/40 (0%)	3/40 (8%)	37/40 (93%)	MAPK14,MAPK1,MAPK3
Adenine and Adenosine Salvage VI	1.84	0.20	0/5 (0%)	0/5 (0%)	1/5 (20%)	4/5 (80%)	ADK
Non-Small Cell Lung Cancer Signaling	1.83	0.05	0/83 (0%)	0/83 (0%)	4/83 (5%)	79/83 (95%)	MAPK1,GRB2,MAPK3,HRAS
Sertoli Cell-Sertoli Cell Junction Signaling	1.82	0.04	0/198 (0%)	0/198 (0%)	7/198 (4%)	191/198 (96%)	PRKACB,MAPK14,PRKAR2B,MAPK1,MAPK3,PRKACA,HRAS
cAMP-mediated signaling	1.81	0.04	0/226 (0%)	0/226 (0%)	8/226 (4%)	218/226 (96%)	PRKACB,GNAS,PRKAR2B,MAPK1,MAPK3,GNAO1,GNAI1,PRKACA
PXR/RXR Activation	1.78	0.04	1/92 (1%)	0/92 (0%)	3/92 (3%)	88/92 (96%)	PRKACB,PRKAR2B,PCK2,PRKACA
Erythropoietin Signaling	1.78	0.05	0/79 (0%)	0/79 (0%)	4/79 (5%)	75/79 (95%)	MAPK1,GRB2,MAPK3,HRAS
Role of MAPK Signaling in the Pathogenesis of Influenza	1.78	0.06	0/71 (0%)	0/71 (0%)	4/71 (6%)	67/71 (94%)	MAPK14,MAPK1,MAPK3,HRAS
Calcium Signaling	1.78	0.03	0/217 (0%)	0/217 (0%)	7/217 (3%)	210/217 (97%)	PRKACB,MYH10,PRKAR2B,MAPK1,MAPK3,PRKACA,ASPH

Ingenuity Canonical Pathways	-log(p-value)	Ratio	Downregulated	No change	Upregulated	No overlap with dataset	Molecules
Huntington's Disease Signaling	1.78	0.03	1/252 (0%)	0/252 (0%)	7/252 (3%)	244/252 (97%)	MAPK1,GRB2,MAPK3,GNAQ,PLCB3,HRAS,CYCS,RASA1
Chondroitin Sulfate Degradation (Metazoa)	1.77	0.09	0/23 (0%)	0/23 (0%)	2/23 (9%)	21/23 (91%)	MGEA5,HEXA
Vitamin-C Transport	1.77	0.09	0/22 (0%)	0/22 (0%)	2/22 (9%)	20/22 (91%)	NXN,TXNRD1
Neurotrophin/TRK Signaling	1.76	0.05	0/76 (0%)	0/76 (0%)	4/76 (5%)	72/76 (95%)	MAPK1,GRB2,MAPK3,HRAS
tRNA Charging	1.75	0.04	2/81 (2%)	0/81 (0%)	1/81 (1%)	78/81 (96%)	PARS2,CARS,MARS2
Netrin Signaling	1.72	0.05	0/58 (0%)	0/58 (0%)	3/58 (5%)	55/58 (95%)	PRKACB,PRKAR2B,PRKACA
CCR5 Signaling in Macrophages	1.72	0.04	0/97 (0%)	0/97 (0%)	4/97 (4%)	93/97 (96%)	GNAS,MAPK14,MAPK1,GNAI1
Renal Cell Carcinoma Signaling	1.72	0.05	0/79 (0%)	0/79 (0%)	4/79 (5%)	75/79 (95%)	MAPK1,GRB2,MAPK3,HRAS
Isoleucine Degradation I	1.71	0.07	1/30 (3%)	0/30 (0%)	1/30 (3%)	28/30 (93%)	ACAT2,EHHADH
Dermatan Sulfate Degradation (Metazoa)	1.71	0.09	0/23 (0%)	0/23 (0%)	2/23 (9%)	21/23 (91%)	MGEA5,HEXA
Mevalonate Pathway I	1.71	0.07	0/29 (0%)	0/29 (0%)	2/29 (7%)	27/29 (93%)	ACAT2,IDI1
IL-3 Signaling	1.70	0.05	0/75 (0%)	0/75 (0%)	4/75 (5%)	71/75 (95%)	MAPK1,GRB2,MAPK3,HRAS
Thyroid Cancer Signaling	1.69	0.07	0/44 (0%)	0/44 (0%)	3/44 (7%)	41/44 (93%)	MAPK1,MAPK3,HRAS
ERK/MAPK Signaling	1.69	0.03	0/211 (0%)	0/211 (0%)	7/211 (3%)	204/211 (97%)	PRKACB,PRKAR2B,MAPK1,GRB2,MAPK3,PRKACA,HRAS
IL-17 Signaling	1.68	0.05	0/75 (0%)	0/75 (0%)	4/75 (5%)	71/75 (95%)	MAPK14,MAPK1,MAPK3,HRAS
Regulation of eIF4 and p70S6K Signaling	1.67	0.03	1/175 (1%)	0/175 (0%)	5/175 (3%)	169/175 (97%)	MAPK14,MAPK1,GRB2,MAPK3,EIF2B5,HRAS
Fc Epsilon RI Signaling	1.66	0.04	0/117 (0%)	0/117 (0%)	5/117 (4%)	112/117 (96%)	MAPK14,MAPK1,GRB2,MAPK3,HRAS
LPS-stimulated MAPK Signaling	1.66	0.05	0/83 (0%)	0/83 (0%)	4/83 (5%)	79/83 (95%)	MAPK14,MAPK1,MAPK3,HRAS
Prolactin Signaling	1.66	0.05	0/84 (0%)	0/84 (0%)	4/84 (5%)	80/84 (95%)	MAPK1,GRB2,MAPK3,HRAS
Oxidative Ethanol Degradation III	1.65	0.05	1/40 (3%)	0/40 (0%)	1/40 (3%)	38/40 (95%)	ACSS1,ACSL1
Melanoma Signaling	1.64	0.06	0/50 (0%)	0/50 (0%)	3/50 (6%)	47/50 (94%)	MAPK1,MAPK3,HRAS
Role of Tissue Factor in Cancer	1.63	0.04	0/130 (0%)	0/130 (0%)	5/130 (4%)	125/130 (96%)	MAPK14,MAPK1,MAPK3,GNAQ,HRAS
Protein Kinase A Signaling	1.62	0.03	2/407 (0%)	0/407 (0%)	9/407 (2%)	396/407 (97%)	PRKACB,H1F0,MYH10,GNAS,PRKAR2B,MAPK1,MAPK3,GNAI1,PRKACA,GNAQ,PLCB3
Aldosterone Signaling in Epithelial Cells	1.62	0.04	1/168 (1%)	0/168 (0%)	5/168 (3%)	162/168 (96%)	MAPK1,MAPK3,HSPH1,PLCB3,DNAJB6,DNAJA1
Glutathione Redox Reactions I	1.60	0.08	1/24 (4%)	0/24 (0%)	1/24 (4%)	22/24 (92%)	GPX3,MGST1
VEGF Family Ligand-Receptor Interactions	1.60	0.05	0/88 (0%)	0/88 (0%)	4/88 (5%)	84/88 (95%)	MAPK1,GRB2,MAPK3,HRAS
Xenobiotic Metabolism Signaling	1.60	0.03	4/304 (1%)	0/304 (0%)	5/304 (2%)	295/304 (97%)	CEL,MGST1,MAPK14,ALDH1L2,MAPK1,MAPK3,GCLC,HRAS,PNPLA6
Colorectal Cancer Metastasis Signaling	1.59	0.03	0/268 (0%)	0/268 (0%)	8/268 (3%)	260/268 (97%)	PRKACB,GNAS,PRKAR2B,MAPK1,GRB2,MAPK3,PRKACA,HRAS
Acute Myeloid Leukemia Signaling	1.59	0.05	0/84 (0%)	0/84 (0%)	4/84 (5%)	80/84 (95%)	MAPK1,GRB2,MAPK3,HRAS
Germ Cell-Sertoli Cell Junction Signaling	1.57	0.04	1/169 (1%)	0/169 (0%)	5/169 (3%)	163/169 (96%)	MAPK14,MAPK1,MAPK3,HRAS,LAMC3,GSN
IL-6 Signaling	1.56	0.04	0/124 (0%)	0/124 (0%)	5/124 (4%)	119/124 (96%)	MAPK14,MAPK1,GRB2,MAPK3,HRAS
Ethanol Degradation IV	1.56	0.07	1/29 (3%)	0/29 (0%)	1/29 (3%)	27/29 (93%)	ACSS1,ACSL1
Regulation of IL-2 Expression in Activated and Anergic T Lymphocytes	1.55	0.04	0/89 (0%)	0/89 (0%)	4/89 (4%)	85/89 (96%)	MAPK1,GRB2,MAPK3,HRAS
14-3-3-mediated Signaling	1.55	0.04	1/121 (1%)	0/121 (0%)	4/121 (3%)	116/121 (96%)	MAPK1,GRB2,MAPK3,PLCB3,HRAS
RhoA Signaling	1.55	0.04	0/122 (0%)	0/122 (0%)	5/122 (4%)	117/122 (96%)	ACTR2,SEPT5,ARPC1A,PFN2,SEPT6
Superpathway of Geranylgeranyldiphosphate Biosynthesis I	1.51	0.05	0/37 (0%)	0/37 (0%)	2/37 (5%)	35/37 (95%)	ACAT2,IDI1
Regulation of Actin-based Motility by Rho	1.50	0.04	0/91 (0%)	0/91 (0%)	4/91 (4%)	87/91 (96%)	ACTR2,ARPC1A,PFN2,GSN
Retinol Biosynthesis	1.49	0.05	2/58 (3%)	0/58 (0%)	1/58 (2%)	55/58 (95%)	CEL,PNLIPRP1,PNPLA6
Prostate Cancer Signaling	1.48	0.04	0/103 (0%)	0/103 (0%)	4/103 (4%)	99/103 (96%)	MAPK1,GRB2,MAPK3,HRAS
Role of Macrophages, Fibroblasts and Endothelial Cells in Rheumatoid Arthritis	1.45	0.03	2/342 (1%)	0/342 (0%)	7/342 (2%)	333/342 (97%)	FN1,MAPK14,MAPK1,MAPK3,GNAO1,GNAQ,PLCB3,HRAS,PRSS1
Salvage Pathways of Pyrimidine Ribonucleotides	1.45	0.04	1/103 (1%)	0/103 (0%)	3/103 (3%)	99/103 (96%)	FAM20B,MAPK1,MAPK3,A1CF
Glucocorticoid Receptor Signaling	1.42	0.03	1/299 (0%)	0/299 (0%)	7/299 (2%)	291/299 (97%)	PRKACB,MAPK14,PCK2,MAPK1,GRB2,MAPK3,PRKACA,HRAS
FAK Signaling	1.42	0.04	0/106 (0%)	0/106 (0%)	4/106 (4%)	102/106 (96%)	MAPK1,GRB2,MAPK3,HRAS
RANK Signaling in Osteoclasts	1.42	0.04	0/97 (0%)	0/97 (0%)	4/97 (4%)	93/97 (96%)	MAPK14,MAPK1,MAPK3,GSN
Neuregulin Signaling	1.40	0.04	0/104 (0%)	0/104 (0%)	4/104 (4%)	100/104 (96%)	MAPK1,GRB2,MAPK3,HRAS
PAK Signaling	1.40	0.04	0/110 (0%)	0/110 (0%)	4/110 (4%)	106/110 (96%)	MAPK1,GRB2,MAPK3,HRAS
Apoptosis Signaling	1.40	0.04	0/100 (0%)	0/100 (0%)	4/100 (4%)	96/100 (96%)	MAPK1,MAPK3,HRAS,CYCS
UVB-Induced MAPK Signaling	1.38	0.05	0/58 (0%)	0/58 (0%)	3/58 (5%)	55/58 (95%)	MAPK14,MAPK1,MAPK3
Phototransduction Pathway	1.38	0.04	0/67 (0%)	0/67 (0%)	3/67 (4%)	64/67 (96%)	PRKACB,PRKAR2B,PRKACA
Methylglyoxal Degradation I	1.37	0.09	0/11 (0%)	0/11 (0%)	1/11 (9%)	10/11 (91%)	HAGH
Glutathione Biosynthesis	1.37	0.09	1/11 (9%)	0/11 (0%)	0/11 (0%)	10/11 (91%)	GCLC
N-acetylglucosamine Degradation I	1.37	0.13	0/8 (0%)	0/8 (0%)	1/8 (13%)	7/8 (88%)	GNPDA2
TCA Cycle II (Eukaryotic)	1.36	0.05	0/41 (0%)	0/41 (0%)	2/41 (5%)	39/41 (95%)	SUCLA2,IDH3G
Role of IL-17A in Arthritis	1.36	0.05	0/64 (0%)	0/64 (0%)	3/64 (5%)	61/64 (95%)	MAPK14,MAPK1,MAPK3
HMGB1 Signaling	1.33	0.04	0/109 (0%)	0/109 (0%)	4/109 (4%)	105/109 (96%)	MAPK14,MAPK1,MAPK3,HRAS
Tumoricidal Function of Hepatic Natural Killer Cells	1.33	0.07	0/27 (0%)	0/27 (0%)	2/27 (7%)	25/27 (93%)	M6PR,CYCS
Toll-like Receptor Signaling	1.32	0.05	0/64 (0%)	0/64 (0%)	3/64 (5%)	61/64 (95%)	ECSIT,MAPK14,MAPK1
Chronic Myeloid Leukemia Signaling	1.31	0.04	0/106 (0%)	0/106 (0%)	4/106 (4%)	102/106 (96%)	MAPK1,GRB2,MAPK3,HRAS
PPAR Signaling	1.31	0.04	0/107 (0%)	0/107 (0%)	4/107 (4%)	103/107 (96%)	MAPK1,GRB2,MAPK3,HRAS
Glioma Signaling	1.30	0.04	0/113 (0%)	0/113 (0%)	4/113 (4%)	109/113 (96%)	MAPK1,GRB2,MAPK3,HRAS

Ingenuity Canonical Pathways	-log(p-value)	Ratio	Downregulated	No change	Upregulated	No overlap with dataset	Molecules
Myc Mediated Apoptosis Signaling	1.28	0.05	0/63 (0%)	0/63 (0%)	3/63 (5%)	60/63 (95%)	GRB2,HRAS,CYCS
Hepatic Cholestasis	1.28	0.03	1/183 (1%)	0/183 (0%)	4/183 (2%)	178/183 (97%)	PRKACB,GNAS,PRKAR2B,PRKACA,ATP8B1
TREM1 Signaling	1.26	0.04	0/75 (0%)	0/75 (0%)	3/75 (4%)	72/75 (96%)	MAPK1,GRB2,MAPK3
Phospholipase C Signaling	1.25	0.03	1/265 (0%)	0/265 (0%)	6/265 (2%)	258/265 (97%)	GNAS,MAPK1,GRB2,MAPK3,GNAQ,PLCB3,HRAS
Uracil Degradation II (Reductive)	1.24	0.09	0/11 (0%)	0/11 (0%)	1/11 (9%)	10/11 (91%)	DPYSL2
Trehalose Degradation II (Trehalase)	1.24	0.11	0/9 (0%)	0/9 (0%)	1/9 (11%)	8/9 (89%)	HK1
Heme Biosynthesis from Uroporphyrinogen-III I	1.24	0.09	0/11 (0%)	0/11 (0%)	1/11 (9%)	10/11 (91%)	CPOX
Thymine Degradation	1.24	0.09	0/11 (0%)	0/11 (0%)	1/11 (9%)	10/11 (91%)	DPYSL2
Arginine Degradation I (Arginase Pathway)	1.24	0.08	0/13 (0%)	0/13 (0%)	1/13 (8%)	12/13 (92%)	OAT
N-acetylglucosamine Degradation II	1.24	0.08	0/12 (0%)	0/12 (0%)	1/12 (8%)	11/12 (92%)	GNPDA2
HGF Signaling	1.23	0.04	0/111 (0%)	0/111 (0%)	4/111 (4%)	107/111 (96%)	MAPK1,GRB2,MAPK3,HRAS
Paxillin Signaling	1.22	0.03	0/117 (0%)	0/117 (0%)	4/117 (3%)	113/117 (97%)	MAPK14,MAPK1,GRB2,HRAS
Telomerase Signaling	1.22	0.04	0/106 (0%)	0/106 (0%)	4/106 (4%)	102/106 (96%)	MAPK1,GRB2,MAPK3,HRAS
HIF1α ± Signaling	1.20	0.04	0/112 (0%)	0/112 (0%)	4/112 (4%)	108/112 (96%)	MAPK14,MAPK1,MAPK3,HRAS
Glioblastoma Multiforme Signaling	1.19	0.03	1/168 (1%)	0/168 (0%)	4/168 (2%)	163/168 (97%)	MAPK1,GRB2,MAPK3,PLCB3,HRAS
CD40 Signaling	1.19	0.04	0/71 (0%)	0/71 (0%)	3/71 (4%)	68/71 (96%)	MAPK14,MAPK1,MAPK3
Estrogen-Dependent Breast Cancer Signaling	1.19	0.04	0/73 (0%)	0/73 (0%)	3/73 (4%)	70/73 (96%)	MAPK1,MAPK3,HRAS
Antiproliferative Role of Somatostatin Receptor 2	1.18	0.04	0/72 (0%)	0/72 (0%)	3/72 (4%)	69/72 (96%)	MAPK1,MAPK3,HRAS
IL-17A Signaling in Airway Cells	1.18	0.04	0/76 (0%)	0/76 (0%)	3/76 (4%)	73/76 (96%)	MAPK14,MAPK1,MAPK3
Integrin Signaling	1.16	0.03	0/208 (0%)	0/208 (0%)	6/208 (3%)	202/208 (97%)	ACTR2,ARPC1A,MAPK1,GRB2,MAPK3,HRAS
Angiopoietin Signaling	1.16	0.04	0/75 (0%)	0/75 (0%)	3/75 (4%)	72/75 (96%)	GRB2,HRAS,RASA1
Role of PI3K/AKT Signaling in the Pathogenesis of Influenza	1.16	0.04	0/76 (0%)	0/76 (0%)	3/76 (4%)	73/76 (96%)	MAPK1,MAPK3,GNAI1
Pyridoxal 5'-phosphate Salvage Pathway	1.16	0.04	0/75 (0%)	0/75 (0%)	3/75 (4%)	72/75 (96%)	FAM20B,MAPK1,MAPK3
Proline Biosynthesis II (from Arginine)	1.15	0.05	0/20 (0%)	0/20 (0%)	1/20 (5%)	19/20 (95%)	OAT
CMP-N-acetylneuraminate Biosynthesis I (Eukaryotes)	1.15	0.06	0/18 (0%)	0/18 (0%)	1/18 (6%)	17/18 (94%)	CMAS
Lysine Degradation II	1.15	0.05	0/19 (0%)	0/19 (0%)	1/19 (5%)	18/19 (95%)	AASS
Trans, trans-farnesyl Diphosphate Biosynthesis	1.15	0.10	0/10 (0%)	0/10 (0%)	1/10 (10%)	9/10 (90%)	IDI1
Phenylalanine Degradation I (Aerobic)	1.15	0.08	0/12 (0%)	0/12 (0%)	1/12 (8%)	11/12 (92%)	QDPR
NGF Signaling	1.15	0.03	0/122 (0%)	0/122 (0%)	4/122 (3%)	118/122 (97%)	MAPK1,GRB2,MAPK3,HRAS
Type I Diabetes Mellitus Signaling	1.14	0.03	0/121 (0%)	0/121 (0%)	4/121 (3%)	117/121 (97%)	MAPK14,MAPK1,HLA-A,CYCS
Tight Junction Signaling	1.13	0.03	0/167 (0%)	0/167 (0%)	5/167 (3%)	162/167 (97%)	PRKACB,MYH10,PRKAR2B,NUDT21,PRKACA
Natural Killer Cell Signaling	1.11	0.03	0/118 (0%)	0/118 (0%)	4/118 (3%)	114/118 (97%)	MAPK1,GRB2,MAPK3,HRAS
Tec Kinase Signaling	1.11	0.03	0/184 (0%)	0/184 (0%)	5/184 (3%)	179/184 (97%)	GNAS,GNAO1,GNAI1,GNAQ,GNAZ
Role of NANOG in Mammalian Embryonic Stem Cell Pluripotency	1.10	0.03	0/118 (0%)	0/118 (0%)	4/118 (3%)	114/118 (97%)	MAPK1,GRB2,MAPK3,HRAS
MIF-mediated Glucocorticoid Regulation	1.08	0.05	0/42 (0%)	0/42 (0%)	2/42 (5%)	40/42 (95%)	MAPK1,MAPK3
Ethanol Degradation II	1.08	0.05	1/43 (2%)	0/43 (0%)	1/43 (2%)	41/43 (95%)	ACSS1,ACSL1
B Cell Receptor Signaling	1.08	0.03	0/175 (0%)	0/175 (0%)	5/175 (3%)	170/175 (97%)	MAPK14,MAPK1,GRB2,MAPK3,HRAS
Arginine Biosynthesis IV	1.07	0.04	0/24 (0%)	0/24 (0%)	1/24 (4%)	23/24 (96%)	OAT
Arginine Degradation VI (Arginase 2 Pathway)	1.07	0.06	0/16 (0%)	0/16 (0%)	1/16 (6%)	15/16 (94%)	OAT
Selenocysteine Biosynthesis II (Archaea and Eukaryotes)	1.07	0.06	0/17 (0%)	0/17 (0%)	1/17 (6%)	16/17 (94%)	SEPHS1
UDP-N-acetyl-D-glucosamine Biosynthesis II	1.07	0.06	1/17 (6%)	0/17 (0%)	0/17 (0%)	16/17 (94%)	UAP1
Ceramide Degradation	1.07	0.07	0/14 (0%)	0/14 (0%)	1/14 (7%)	13/14 (93%)	ASAH1
Zymosterol Biosynthesis	1.07	0.05	0/22 (0%)	0/22 (0%)	1/22 (5%)	21/22 (95%)	NSDHL
Tyrosine Degradation I	1.07	0.07	0/15 (0%)	0/15 (0%)	1/15 (7%)	14/15 (93%)	FAH
GDP-mannose Biosynthesis	1.07	0.08	0/13 (0%)	0/13 (0%)	1/13 (8%)	12/13 (92%)	GPI
Inhibition of Angiogenesis by TSP1	1.06	0.05	0/42 (0%)	0/42 (0%)	2/42 (5%)	40/42 (95%)	MAPK14,MAPK1
Type II Diabetes Mellitus Signaling	1.05	0.02	0/171 (0%)	0/171 (0%)	4/171 (2%)	167/171 (98%)	MAPK1,MAPK3,ABCC8,ACSL1
PTEN Signaling	1.04	0.03	0/138 (0%)	0/138 (0%)	4/138 (3%)	134/138 (97%)	MAPK1,GRB2,MAPK3,HRAS
Coagulation System	1.04	0.05	0/38 (0%)	0/38 (0%)	2/38 (5%)	36/38 (95%)	KNG1,PLG
Phosphatidylcholine Biosynthesis I	1.01	0.06	0/17 (0%)	0/17 (0%)	1/17 (6%)	16/17 (94%)	PCYT1A
PI3K/AKT Signaling	0.98	0.03	0/152 (0%)	0/152 (0%)	4/152 (3%)	148/152 (97%)	MAPK1,GRB2,MAPK3,HRAS
April Mediated Signaling	0.98	0.05	0/44 (0%)	0/44 (0%)	2/44 (5%)	42/44 (95%)	MAPK14,MAPK1
Docosahexaenoic Acid (DHA) Signaling	0.96	0.04	0/50 (0%)	0/50 (0%)	2/50 (4%)	48/50 (96%)	SERPINF1,CYCS
Sphingosine and Sphingosine-1-phosphate Metabolism	0.96	0.05	0/21 (0%)	0/21 (0%)	1/21 (5%)	20/21 (95%)	ASAH1
Sucrose Degradation V (Mammalian)	0.96	0.05	0/19 (0%)	0/19 (0%)	1/19 (5%)	18/19 (95%)	ALDOC
Citrulline Biosynthesis	0.96	0.04	0/26 (0%)	0/26 (0%)	1/26 (4%)	25/26 (96%)	OAT
Glycoaminoglycan-protein Linkage Region Biosynthesis	0.96	0.06	0/16 (0%)	0/16 (0%)	1/16 (6%)	15/16 (94%)	B3GAT3
GDP-glucose Biosynthesis	0.96	0.05	0/19 (0%)	0/19 (0%)	1/19 (5%)	18/19 (95%)	HK1
Ceramide Signaling	0.95	0.03	0/91 (0%)	0/91 (0%)	3/91 (3%)	88/91 (97%)	MAPK3,HRAS,CYCS
Role of PKR in Interferon Induction and Antiviral Response	0.94	0.04	0/49 (0%)	0/49 (0%)	2/49 (4%)	47/49 (96%)	MAPK14,CYCS

Ingenuity Canonical Pathways	-log(p-value)	Ratio	Downregulated	No change	Upregulated	No overlap with dataset	Molecules
B Cell Activating Factor Signaling	0.94	0.04	0/46 (0%)	0/46 (0%)	2/46 (4%)	44/46 (96%)	MAPK14,MAPK1
PI3K Signaling in B Lymphocytes	0.93	0.03	1/143 (1%)	0/143 (0%)	3/143 (2%)	139/143 (97%)	MAPK1,MAPK3,PLCB3,HRAS
Dendritic Cell Maturation	0.92	0.02	1/211 (0%)	0/211 (0%)	4/211 (2%)	206/211 (98%)	MAPK14,MAPK1,HLA-A,MAPK3,PLCB3
MIF Regulation of Innate Immunity	0.92	0.04	0/52 (0%)	0/52 (0%)	2/52 (4%)	50/52 (96%)	MAPK1,MAPK3
FcÎ³RIIB Signaling in B Lymphocytes	0.92	0.03	0/64 (0%)	0/64 (0%)	2/64 (3%)	62/64 (97%)	GRB2,HRAS
Assembly of RNA Polymerase I Complex	0.91	0.08	0/13 (0%)	0/13 (0%)	1/13 (8%)	12/13 (92%)	POLR1C
Glucose and Glucose-1-phosphate Degradation	0.91	0.04	0/24 (0%)	0/24 (0%)	1/24 (4%)	23/24 (96%)	HK1
EIF2 Signaling	0.88	0.02	1/201 (0%)	0/201 (0%)	4/201 (2%)	196/201 (98%)	MAPK1,GRB2,MAPK3,EIF2B5,HRAS
iNOS Signaling	0.87	0.04	0/53 (0%)	0/53 (0%)	2/53 (4%)	51/53 (96%)	MAPK14,MAPK1
Heme Biosynthesis II	0.87	0.04	0/24 (0%)	0/24 (0%)	1/24 (4%)	23/24 (96%)	CPOX
CTLA4 Signaling in Cytotoxic T Lymphocytes	0.86	0.03	0/96 (0%)	0/96 (0%)	3/96 (3%)	93/96 (97%)	GRB2,HLA-A,AP1M1
IL-8 Signaling	0.86	0.02	0/225 (0%)	0/225 (0%)	5/225 (2%)	220/225 (98%)	GNAS,MAPK1,MAPK3,GNAI1,HRAS
Role of IL-17F in Allergic Inflammatory Airway Diseases	0.86	0.04	0/48 (0%)	0/48 (0%)	2/48 (4%)	46/48 (96%)	MAPK1,MAPK3
Bladder Cancer Signaling	0.85	0.03	0/97 (0%)	0/97 (0%)	3/97 (3%)	94/97 (97%)	MAPK1,MAPK3,HRAS
Aryl Hydrocarbon Receptor Signaling	0.83	0.02	2/171 (1%)	0/171 (0%)	2/171 (1%)	167/171 (98%)	MGST1,ALDH1L2,MAPK1,MAPK3
Ketogenesis	0.83	0.05	0/21 (0%)	0/21 (0%)	1/21 (5%)	20/21 (95%)	ACAT2
Cleavage and Polyadenylation of Pre-mRNA	0.79	0.08	0/13 (0%)	0/13 (0%)	1/13 (8%)	12/13 (92%)	NUDT21
Guanosine Nucleotides Degradation III	0.79	0.05	1/22 (5%)	0/22 (0%)	0/22 (0%)	21/22 (95%)	AOX1
Epithelial Adherens Junction Signaling	0.78	0.03	0/154 (0%)	0/154 (0%)	4/154 (3%)	150/154 (97%)	MYH10,ACTR2,ARPC1A,HRAS
Role of Pattern Recognition Receptors in Recognition of Bacteria and Viruses	0.78	0.03	0/109 (0%)	0/109 (0%)	3/109 (3%)	106/109 (97%)	MAPK1,MAPK3,C1QC
Protein Ubiquitination Pathway	0.77	0.02	0/270 (0%)	0/270 (0%)	6/270 (2%)	264/270 (98%)	UCHL1,HLA-A,HSPH1,PSMD14,DNAJB6,DNAJA1
Assembly of RNA Polymerase III Complex	0.76	0.06	0/16 (0%)	0/16 (0%)	1/16 (6%)	15/16 (94%)	SF3A1
Fatty Acid Activation	0.76	0.05	0/19 (0%)	0/19 (0%)	1/19 (5%)	18/19 (95%)	ACSL1
Choline Biosynthesis III	0.76	0.05	0/22 (0%)	0/22 (0%)	1/22 (5%)	21/22 (95%)	PCYT1A
Cholesterol Biosynthesis I	0.76	0.03	0/40 (0%)	0/40 (0%)	1/40 (3%)	39/40 (98%)	NSDHL
Urate Biosynthesis/Inosine 5'-phosphate Degradation	0.76	0.05	1/22 (5%)	0/22 (0%)	0/22 (0%)	21/22 (95%)	AOX1
Bile Acid Biosynthesis, Neutral Pathway	0.76	0.02	0/58 (0%)	0/58 (0%)	1/58 (2%)	57/58 (98%)	AMACR
Cholesterol Biosynthesis II (via 24,25-dihydrolanosterol)	0.76	0.03	0/40 (0%)	0/40 (0%)	1/40 (3%)	39/40 (98%)	NSDHL
Cholesterol Biosynthesis III (via Desmosterol)	0.76	0.03	0/40 (0%)	0/40 (0%)	1/40 (3%)	39/40 (98%)	NSDHL
Nicotine Degradation III	0.76	0.03	1/73 (1%)	0/73 (0%)	1/73 (1%)	71/73 (97%)	B3GAT3,AOX1
Superpathway of Citrulline Metabolism	0.73	0.03	0/38 (0%)	0/38 (0%)	1/38 (3%)	37/38 (97%)	OAT
Colanic Acid Building Blocks Biosynthesis	0.73	0.03	0/36 (0%)	0/36 (0%)	1/36 (3%)	35/36 (97%)	GPI
Mitochondrial Dysfunction	0.73	0.02	0/201 (0%)	0/201 (0%)	4/201 (2%)	197/201 (98%)	SURF1,NDUFS6,DHODH,CYCS
nNOS Signaling in Skeletal Muscle Cells	0.70	0.03	1/31 (3%)	0/31 (0%)	0/31 (0%)	30/31 (97%)	DMD
CDP-diacylglycerol Biosynthesis I	0.70	0.04	1/27 (4%)	0/27 (0%)	0/27 (0%)	26/27 (96%)	LCLAT1
Adenosine Nucleotides Degradation II	0.70	0.04	1/26 (4%)	0/26 (0%)	0/26 (0%)	25/26 (96%)	AOX1
Pancreatic Adenocarcinoma Signaling	0.69	0.02	0/128 (0%)	0/128 (0%)	3/128 (2%)	125/128 (98%)	MAPK1,GRB2,MAPK3
Granzyme B Signaling	0.68	0.06	0/18 (0%)	0/18 (0%)	1/18 (6%)	17/18 (94%)	CYCS
Phosphatidylglycerol Biosynthesis II (Non-plastidic)	0.66	0.03	1/33 (3%)	0/33 (0%)	0/33 (0%)	32/33 (97%)	LCLAT1
Mitochondrial L-carnitine Shuttle Pathway	0.66	0.05	0/22 (0%)	0/22 (0%)	1/22 (5%)	21/22 (95%)	ACSL1
Nicotine Degradation II	0.65	0.02	1/85 (1%)	0/85 (0%)	1/85 (1%)	83/85 (98%)	B3GAT3,AOX1
Valine Degradation I	0.63	0.03	1/35 (3%)	0/35 (0%)	0/35 (0%)	34/35 (97%)	EHHADH
Purine Nucleotides Degradation II (Aerobic)	0.63	0.03	1/35 (3%)	0/35 (0%)	0/35 (0%)	34/35 (97%)	AOX1
D-myo-inositol (1,4,5)-trisphosphate Degradation	0.63	0.04	0/23 (0%)	0/23 (0%)	1/23 (4%)	22/23 (96%)	PMPCA
ERK5 Signaling	0.62	0.03	0/68 (0%)	0/68 (0%)	2/68 (3%)	66/68 (97%)	GNAQ,HRAS
CD28 Signaling in T Helper Cells	0.62	0.02	0/136 (0%)	0/136 (0%)	3/136 (2%)	133/136 (98%)	ACTR2,ARPC1A,GRB2
Granzyme A Signaling	0.61	0.05	1/20 (5%)	0/20 (0%)	0/20 (0%)	19/20 (95%)	H1F0
1D-myo-inositol Hexakisphosphate Biosynthesis II (Mammalian)	0.61	0.04	0/28 (0%)	0/28 (0%)	1/28 (4%)	27/28 (96%)	PMPCA
D-myo-inositol (1,3,4)-trisphosphate Biosynthesis	0.61	0.04	0/25 (0%)	0/25 (0%)	1/25 (4%)	24/25 (96%)	PMPCA
DNA Methylation and Transcriptional Repression Signaling	0.59	0.04	0/23 (0%)	0/23 (0%)	1/23 (4%)	22/23 (96%)	RBBP4
Retinoic acid Mediated Apoptosis Signaling	0.59	0.03	0/73 (0%)	0/73 (0%)	2/73 (3%)	71/73 (97%)	DAP3,CYCS
IL-10 Signaling	0.58	0.03	0/78 (0%)	0/78 (0%)	2/78 (3%)	76/78 (97%)	MAPK14,MAPK1
Remodeling of Epithelial Adherens Junctions	0.58	0.03	0/70 (0%)	0/70 (0%)	2/70 (3%)	68/70 (97%)	ACTR2,ARPC1A
Growth Hormone Signaling	0.58	0.03	0/78 (0%)	0/78 (0%)	2/78 (3%)	76/78 (97%)	MAPK1,MAPK3
Regulation of the Epithelial-Mesenchymal Transition Pathway	0.57	0.02	0/196 (0%)	0/196 (0%)	4/196 (2%)	192/196 (98%)	MAPK1,GRB2,MAPK3,HRAS
Clathrin-mediated Endocytosis Signaling	0.55	0.02	0/198 (0%)	0/198 (0%)	4/198 (2%)	194/198 (98%)	ACTR2,ARPC1A,GRB2,PICALM
Superpathway of Inositol Phosphate Compounds	0.54	0.02	1/230 (0%)	0/230 (0%)	3/230 (1%)	226/230 (98%)	NUDT3,PLCB3,RASA1,PMPCA
ILK Signaling	0.54	0.02	0/205 (0%)	0/205 (0%)	4/205 (2%)	201/205 (98%)	MYH10,FN1,MAPK1,MAPK3
IL-4 Signaling	0.53	0.03	0/80 (0%)	0/80 (0%)	2/80 (3%)	78/80 (98%)	GRB2,HRAS
Superpathway of D-myo-inositol (1,4,5)-trisphosphate Metabolism	0.53	0.03	0/33 (0%)	0/33 (0%)	1/33 (3%)	32/33 (97%)	PMPCA

Ingenuity Canonical Pathways	-log(p-value)	Ratio	Downregulated	No change	Upregulated	No overlap with dataset	Molecules
HER-2 Signaling in Breast Cancer	0.52	0.02	0/82 (0%)	0/82 (0%)	2/82 (2%)	80/82 (98%)	GRB2,HRAS
IL-12 Signaling and Production in Macrophages	0.51	0.02	0/157 (0%)	0/157 (0%)	3/157 (2%)	154/157 (98%)	MAPK14,MAPK1,MAPK3
Antiproliferative Role of TOB in T Cell Signaling	0.50	0.04	0/26 (0%)	0/26 (0%)	1/26 (4%)	25/26 (96%)	MAPK1
D-myo-inositol-5-phosphate Metabolism	0.49	0.02	1/159 (1%)	0/159 (0%)	2/159 (1%)	156/159 (98%)	NUDT3,PLCB3,RASA1
D-myo-inositol (1,4,5)-Trisphosphate Biosynthesis	0.49	0.03	1/38 (3%)	0/38 (0%)	0/38 (0%)	37/38 (97%)	PLCB3
Cytotoxic T Lymphocyte-mediated Apoptosis of Target Cells	0.48	0.02	0/88 (0%)	0/88 (0%)	2/88 (2%)	86/88 (98%)	HLA-A,CYCS
Intrinsic Prothrombin Activation Pathway	0.47	0.03	0/37 (0%)	0/37 (0%)	1/37 (3%)	36/37 (97%)	KNG1
Glutathione-mediated Detoxification	0.47	0.02	1/44 (2%)	0/44 (0%)	0/44 (0%)	43/44 (98%)	MGST1
TR/RXR Activation	0.45	0.02	0/109 (0%)	0/109 (0%)	2/109 (2%)	107/109 (98%)	PFKP,ME1
Role of p14/p19ARF in Tumor Suppression	0.45	0.03	0/35 (0%)	0/35 (0%)	1/35 (3%)	34/35 (97%)	SF3A1
Virus Entry via Endocytic Pathways	0.43	0.02	0/101 (0%)	0/101 (0%)	2/101 (2%)	99/101 (98%)	HLA-A,HRAS
Thyroid Hormone Metabolism II (via Conjugation and/or Degradation)	0.43	0.02	0/53 (0%)	0/53 (0%)	1/53 (2%)	52/53 (98%)	B3GAT3
TWEAK Signaling	0.42	0.03	0/39 (0%)	0/39 (0%)	1/39 (3%)	38/39 (97%)	CYCS
Triacylglycerol Biosynthesis	0.42	0.02	1/46 (2%)	0/46 (0%)	0/46 (0%)	45/46 (98%)	LCLAT1
Serotonin Receptor Signaling	0.42	0.02	0/49 (0%)	0/49 (0%)	1/49 (2%)	48/49 (98%)	QDPR
Role of Osteoblasts, Osteoclasts and Chondrocytes in Rheumatoid Arthritis	0.40	0.02	0/249 (0%)	0/249 (0%)	4/249 (2%)	245/249 (98%)	MAPK14,MAPK1,MAPK3,GSN
Stearate Biosynthesis I (Animals)	0.40	0.02	0/49 (0%)	0/49 (0%)	1/49 (2%)	48/49 (98%)	ACSL1
LPS/IL-1 Mediated Inhibition of RXR Function	0.40	0.02	2/245 (1%)	0/245 (0%)	2/245 (1%)	241/245 (98%)	ECSIT,MGST1,ALDH1L2,ACSL1
SAPK/JNK Signaling	0.39	0.02	0/105 (0%)	0/105 (0%)	2/105 (2%)	103/105 (98%)	GRB2,HRAS
Antigen Presentation Pathway	0.38	0.02	0/42 (0%)	0/42 (0%)	1/42 (2%)	41/42 (98%)	HLA-A
NF-ĸB Signaling	0.36	0.02	0/174 (0%)	0/174 (0%)	3/174 (2%)	171/174 (98%)	PRKACB,PRKACA,HRAS
Production of Nitric Oxide and Reactive Oxygen Species in Macrophages	0.31	0.01	0/212 (0%)	0/212 (0%)	3/212 (1%)	209/212 (99%)	MAPK14,MAPK1,MAPK3
Graft-versus-Host Disease Signaling	0.31	0.02	0/51 (0%)	0/51 (0%)	1/51 (2%)	50/51 (98%)	HLA-A
Autoimmune Thyroid Disease Signaling	0.30	0.02	0/62 (0%)	0/62 (0%)	1/62 (2%)	61/62 (98%)	HLA-A
TNFR1 Signaling	0.30	0.02	0/54 (0%)	0/54 (0%)	1/54 (2%)	53/54 (98%)	CYCS
nNOS Signaling in Neurons	0.30	0.02	0/52 (0%)	0/52 (0%)	1/52 (2%)	51/52 (98%)	PFKM
Agranulocyte Adhesion and Diapedesis	0.30	0.02	0/191 (0%)	0/191 (0%)	3/191 (2%)	188/191 (98%)	MYH10,FN1,GNAI1
CD27 Signaling in Lymphocytes	0.28	0.02	0/59 (0%)	0/59 (0%)	1/59 (2%)	58/59 (98%)	CYCS
D-myo-inositol (1,4,5,6)-Tetrakisphosphate Biosynthesis	0.28	0.01	0/142 (0%)	0/142 (0%)	2/142 (1%)	140/142 (99%)	NUDT3,RASA1
D-myo-inositol (3,4,5,6)-tetrakisphosphate Biosynthesis	0.28	0.01	0/142 (0%)	0/142 (0%)	2/142 (1%)	140/142 (99%)	NUDT3,RASA1
mTOR Signaling	0.28	0.01	0/213 (0%)	0/213 (0%)	3/213 (1%)	210/213 (99%)	MAPK1,MAPK3,HRAS
Leukocyte Extravasation Signaling	0.26	0.01	0/210 (0%)	0/210 (0%)	3/210 (1%)	207/210 (99%)	MAPK14,MAPK1,GNAI1
Nur77 Signaling in T Lymphocytes	0.26	0.02	0/64 (0%)	0/64 (0%)	1/64 (2%)	63/64 (98%)	CYCS
Melatonin Degradation I	0.26	0.02	0/66 (0%)	0/66 (0%)	1/66 (2%)	65/66 (98%)	B3GAT3
Chondroitin Sulfate Biosynthesis	0.26	0.01	0/72 (0%)	0/72 (0%)	1/72 (1%)	71/72 (99%)	B3GAT3
Phospholipases	0.25	0.01	1/67 (1%)	0/67 (0%)	0/67 (0%)	66/67 (99%)	PLCB3
Dermatan Sulfate Biosynthesis	0.25	0.01	0/73 (0%)	0/73 (0%)	1/73 (1%)	72/73 (99%)	B3GAT3
Heparan Sulfate Biosynthesis	0.24	0.01	0/75 (0%)	0/75 (0%)	1/75 (1%)	74/75 (99%)	B3GAT3
Death Receptor Signaling	0.24	0.01	0/68 (0%)	0/68 (0%)	1/68 (1%)	67/68 (99%)	CYCS
Induction of Apoptosis by HIV1	0.24	0.01	0/67 (0%)	0/67 (0%)	1/67 (1%)	66/67 (99%)	CYCS
ATM Signaling	0.23	0.02	0/66 (0%)	0/66 (0%)	1/66 (2%)	65/66 (98%)	MAPK14
Superpathway of Melatonin Degradation	0.23	0.01	0/81 (0%)	0/81 (0%)	1/81 (1%)	80/81 (99%)	B3GAT3
Serotonin Degradation	0.23	0.01	0/78 (0%)	0/78 (0%)	1/78 (1%)	77/78 (99%)	B3GAT3
Macropinocytosis Signaling	0.20	0.01	0/77 (0%)	0/77 (0%)	1/77 (1%)	76/77 (99%)	HRAS

Supplementary Table 4 Validation of Seven Proteins Expressed in PNETs by Immunohistochemical Staining and Western blot

Protein	Insulinoma		Non-insulinoma		PNET	
	tumoral tissues	peritumoral tissue	tumoral tissues	peritumoral tissue	tumoral tissues	peritumoral tissue
UCH-L1	22/37 = 59%	1/23 = 4%	5/15 = 33%	0/8 = 0%	27/52 = 52%	1/32 = 3%
MAP1B	25/33 = 76%	0/23 = 0%	7/15 = 47%	0/4 = 0%	32/48 = 67%	0/27 = 0%
MAP2	17/33 = 52%	1/23 = 4%	6/15 = 40%	0/4 = 0%	23/48 = 48%	1/27 = 4%
VCAN	20/25 = 80%	0/13 = 0	10/15 = 67%	0/6 = 0	30/40 = 75%	0/19 = 0%
PDX-1	36/41 = 88%	9/35 = 26%	0/6 = 0	0/6 = 0	36/47 = 77%	9/41 = 22%
CaSR	27/29 = 93%	12/25= 48%				
CDK4	36/37 = 97%	20/25 = 80%	15/15 =100%	5/5 = 100%	51/52 = 98%	25/30 = 83%

Supplementary Table 5 Correlation of Clinicopathological Characteristics with Expression of UCH-L1

Clinicopathological Features		Expression of UCH-L1		
		Present (%)	Absent (%)	P value
**Insulinomas (n=154)**		83(53.9)	71(46.1)	
Ki-67(n=126)	≦2%	52(52.0)	48(48.0)	0.867
	＞2%	14(53.8)	12(46.2)	
metastasis (n=151)	Yes	3 (37.5)	5(62.5)	0.327
	No	79(55.2)	64(44.8)	
stage (n=150)	I	47(56.0)	37(44.0)	0.926
	II	31(54.4)	26(45.6)	
	III	1(50)	1(50)	
	IV	3 (42.9)	4(57.1)	
overall survival (n=101)		56(55.4)	45(44.6)	0.179
death (n=5)		1 (20.0)	4(80.0)	
disease free survival (n=97)		55(56.7)	42(43.3)	0.047

survival with disease or death (n=7)		1(14.3)	6(85.7)	
**All PNETs (n=314)**		137(43.6)	177(56.4)	
location(n=292)	pancreatic head	60(47.2)	67(52.8)	0.473
	body/tail	71(43.0)	94(57.0)	
Ki-67 (n=234)	≦2%	75(43.6)	97(56.4)	0.654
	＞2%	25(40.3)	37(59.7)	
metastasis(n=314)	Yes	31(37.8)	51(62.2)	0.216
	No	106(45.7)	126(54.3)	
size [range](cm) (n=302)		2[0.7-15]	3 [0.8-17]	0.024
**Patients with PNETs(n=306)**		135(44.1)	171(55.9)	
age at diagnosis (year)(n=305)		47(18-85)	49.5(15-84)	0.078
gender (n=305)	male	47(36.4)	82(63.6)	0.024
	female	87(49.4)	89(50.6)	
grade (n=250)	G1	57(40.7)	83(59.3)	0.303
	G2	48(46.2)	56(53.8)	

	G3	1(16.7)	5(83.3)	
stage (n=305)	I	50(52.6)	45(47.4)	0.093
	II a	45(45.9)	53(54.1)	
	II b	11(31.4)	24(68.6)	
	III	14(46.7)	16(53.3)	
	IV	14(29.8)	33(70.2)	
recurrence (n=235)	No	80(47.7)	92(52.3)	0.071
	Yes	21(30.5)	42(69.5)	
overall survival (n=199)		86(43.2)	113(56.8)	0.466
death (n=38)		14(36.8)	24(63.2)	
disease free survival (n=170)		80(47.1)	90(52.9)	0.013
survival with disease or death (n=65)		19(29.2)	46(70.8)	

Supplementary Table 6-a Cox Models of Overall Survival

Variable N=214	Model A		Variable N=209	Model B	
	HR (95% CI)	*P* value		HR (95% CI)	*P* value
Age (year)	0.991 (0.961-1.022)	0.567	Age (year)	0.983 (0.952-1.014)	0.281
Gender	0.952 (0.465-1.952)	0.894	Gender	1.091 (0.551-2.161)	0.802
Grade	2.685 (1.250-5.768)	0.011	Grade	2.696 (1.291-5.629)	0.008
Stage	3.118 (2.086-4.662)	2.99E-8	Stage	2.652 (1.766-3.982)	2.55E-6
UCH-L1 [a]	0.757 (0.361-1.591)	0.463	UCH-L1 and α-internexin [b]	0.141 (0.018-1.088)	0.060

Supplementary Table 6-b Cox Models of Disease-free Survival

Variable N=200	Model A		Variable N=194	Model B	
	HR (95% CI)	*P* value		HR (95% CI)	*P* value
Age (year)	0.982 (0.960-1.004)	0.102	Age (year)	0.975 (0.954-0.997)	0.027
Gender	0.895 (0.488-1.642)	0.720	Gender	1.135 (0.624-2.065)	0.678
Grade	3.978 (2.114-7.484)	1.86E-5	Grade	3.323 (1.836-6.016)	7.33E-5
Stage	3.791 (2.721-5.283)	1.04E-13	Stage	3.327 (2.383-4.644)	1.71E-12
UCH-L1 [a]	0.460 (0.243-0.873)	0.018	UCH-L1 and α-internexin [b]	0.215 (0.064-0.716)	0.012

[a] UCH-L1 means positive expression of Ubiquitin C-terminal Hydrolase-L1

[b] UCH-L1 and α-internexin means positive expression of both Ubiquitin C-terminal Hydrolase-L1 and α-internexin

Supplementary Table 7 Cox Models of Disease Free Survival in Patients with Stage II and III

Variable n=116	HR (95% CI)	*P* value
Age(year)	0.987 (0.961-1.013)	0.319
Gender	0.797 (0.356-1.785)	0.582
Grade	7.371 (2.740-19.831)	7.61E-5
Size	1.109 (0.999-1.231)	0.053
Metastasis	0.280 (0.128-0.612)	0.001
UCH-L1 and α-internexin [a]	0.167 (0.038-0.731)	0.017

[a] UCH-L1 and α-internexin means positive expression of both Ubiquitin C-terminal Hydrolase-L1 and α-internexin protein

Supplementary Table 8-a   Cox Models in Non-Insulinoma

Variable N=114	OS		Variable N=104	DFS	
	HR (95% CI)	*P* value		HR (95% CI)	*P* value
Age (year)	0.975 (0.939-1.011)	0.169	Age (year)	0.973 (0.948-0.997)	0.031
Gender	0.977 (0.459-2.080)	0.953	Gender	1.071 (0.572-2.004)	0.831
Grade	2.939 (1.303-6.630)	0.009	Grade	3.100 (1.676-5.732)	3.10E-4
Stage	1.890 (1.209-2.955)	0.005	Stage	2.601 (1.798-3.762)	3.86E-7
UCH-L1 and α-internexin [b]	0.214 (0.028-1.617)	0.135	UCH-L1 and α-internexin [b]	0.419 (0.128-1.369)	0.150

Supplementary Table 8-b Cox Models in Functional PNETs

Variable N=106	DFS	
	HR (95% CI)	*P* value
Age (year)	0.957 (0.919-0.996)	0.033
Gender	1.171 (0.410-3.349)	0.768
Grade	3.847 (0.803-18.431)	0.092
Stage	4.152 (2.252-7.657)	5.14E-6
UCH-L1 and α-internexin [b]	0.180 (0.019-1.662)	0.130

Supplementary Table 8-c Cox Models in Non-functional PNETs

Variable N=93	OS		Variable N=87	DFS	
	HR (95% CI)	*P* value		HR (95% CI)	*P* value
Age (year)	0.969 (0.933-1.006)	0.101	Age (year)	0.984 (0.957-1.012)	0.258
Gender	2.141 (0.877-5.225)	0.094	Gender	1.031 (0.489-2.175)	0.935
Grade	3.346 (1.551-7.219)	0.002	Grade	3.070 (1.600-5.891)	0.001
Stage	2.577 (1.556-4.269)	2.37E-4	Stage	2.982 (1.897-4.686)	2.18E-6
UCH-L1 and α-internexin [b]	0.194 (0.025-1.499)	0.116	UCH-L1 and α-internexin [b]	0.283 (0.067-1.202)	0.087

[b] UCH-L1 and α-internexin means positive expression of both Ubiquitin C-terminal Hydrolase-L1 and α-internexin

Supplementary Table S9 Diagram of Sample Numbers

1. Proteomic Analysis (4 insulinoma tissues and 4 paired peri-tumoral tissues)

2. Validating the expression of 7 proteins by IHC and Western blot

Protein	IHC, n		Western blot, n	
	tumoral tissues	peritumoral tissue	tumoral tissues	Peritumoral or normal tissue
UCH-L1	52	32	10	4
MAP1B	48	27		
MAP2	48	27		
VCAN	40	19		
PDX-1	47	41		
CaSR	29	25	10	4
CDK4	52	30	10	4

3. Methylation-specific PCR (MSP) and Bisulfate Sequencing
(21 fresh frozen tumoral tissues, 9 paired peritumoral tissues and 3 normal pancreatic tissues)

4. UCH-L1 expression

	PNETs	Patients with PNETs	Followed-up patients
All	314	306 [a]	247
Collective I	170	164 [b]	117

5. Both UCH-L1 and INX Expression

	PNETs	Patients with PNETs	Followed-up patients
Two cohorts	290	283 [c]	239
Collective I	155	150 [d]	117
Collective II	135	133 [e]	122

[a] 1 patient has 1 primary tumor and 2 metastatic tumors,
6 patients have 1 primary tumor and 1 metastatic tumor;
[b] 1 patient has 1 primary tumor and 2 metastatic tumors, 4 patients have 1 primary tumor and 1 metastatic tumor;
[c] 1 patient has 1 primary tumor and 2 metastatic tumors, 5 patients have 1 primary tumor and 1 metastatic tumor;
[d] 1 patient has 1 primary tumor and 2 metastatic tumors, 3 patients have 1 primary tumor and 1 metastatic tumor;
[e] 2 patients have 1 primary tumor and 1 metastatic tumor.
